# Targeting Mettl8-Tcf1 axis promotes CD8^+^ T_PEX_ differentiation and antitumor immunity

**DOI:** 10.1084/jem.20250424

**Published:** 2026-03-27

**Authors:** Jiaxi Song, Dan Cui, Jiabao Wang, Xuewei Qi, Jiaxin Ma, Qing Liu, Jing Yang, Haoyu Yu, Lilin Ye, Qizhao Huang, Jia Li, Fengyin Li

**Affiliations:** 1 State Key Laboratory of Immune Response and Immunotherapy, School of Basic Medical Sciences, Division of Life Sciences and Medicine, University of Science and Technology of China, Hefei, China; 2 State Key Laboratory of Respiratory Disease, National Clinical Research Center for Respiratory Disease, National Center for Respiratory Medicine, Joint International Research Laboratory of Respiratory Health, Guangdong Basic Research Center of Excellence for Respiratory Medicine, Guangzhou Institute of Respiratory Health, the First Affiliated Hospital of Guangzhou Medical University, Guangzhou, China; 3 Guangzhou National Laboratory, Guangzhou, China; 4 China-Portugal Artificial Intelligence and Public Health Technologies Joint Laboratory, Guangdong-Hong Kong-Macao Joint Laboratory of Respiratory Infectious Diseases, Guangdong Provincial Key Laboratory of Respiratory Disease Research, Guangzhou Medical University, Guangzhou, China; 5 Institute of Immunological Innovation and Translation, Chongqing Medical University, Chongqing, China; 6 Changping Laboratory, Beijing, China; 7 Department of Hepatobiliary Surgery, The First Affiliated Hospital, Institute of Immunological Innovation and Translation, Chongqing Medical University, Chongqing, China

## Abstract

CD8^+^ T cell exhaustion represents a major obstacle to effective cancer immunotherapy. While stem-like progenitor exhausted T (T_PEX_) cells can differentiate into intermediate (Int-T_EX_) and terminally exhausted (T_EX_) subsets, the epigenetic regulation of this process is unclear. We identify the RNA methyltransferase Mettl8 as a critical regulator, with expression significantly higher in T_PEX_ than in T_EX_ subsets. In anti–PD-1 responding non-small cell lung cancer patients, Mettl8 and the stemness factor *TCF7* were downregulated. In murine models, Mettl8 deletion restrained tumor progression by driving T_PEX_ differentiation into effective Int-T_EX_ cells. Mechanistically, Mettl8 stabilizes *Tcf7* mRNA via m^3^C modification and enhances Tcf1 protein expression. Additionally, Mettl8 interacts with Tcf1 to facilitate chromatin looping at the *Tox* locus, maintaining T_PEX_ stemness. Pharmacological Mettl8 inhibition promoted T_PEX_-to-Int-T_EX_ differentiation and tumor control. Combining this inhibition with anti–PD-1 therapy yielded synergistic efficacy. Our findings establish Mettl8 as a pivotal regulator of T_PEX_ fate and a promising therapeutic target for enhancing immunotherapy.

## Teaser

The study reveals that Mettl8 maintains stem-like T_PEX_ cells by stabilizing Tcf1 via m^3^C modification and facilitating chromatin looping at the Tox locus. Its deletion or inhibition drives T_PEX_ differentiation into effective Int-T_EX_ cells, restraining tumor growth and synergizing with anti–PD-1 therapy, thus presenting a promising immunotherapeutic target.

## Introduction

Over recent decades, extensive research has identified CD8^+^ T cell exhaustion as a major contributor to dysfunction, impairing the effective control of chronic viral infections and tumors. This exhaustion is not an abrupt event but rather a complex, dynamic process. Activated CD8^+^ T cells initially exhibit low effector functions, which gradually increase to a peak, followed by a decline that ultimately leads to dysfunction (Liu et al., 2019; Rahim et al., 2023; Schietinger et al., 2016).

Throughout this progression, significant changes occur in transcriptional profiles and surface marker expression. These include reduced levels of Tcf1, CXCR5, Ly108, and XCL1, coupled with increased expression of Tim3, CD39, CX3CR1, and CD101 (He et al., 2016; Zhou et al., 2023). Tcf1-expressing, stem-like progenitor-exhausted CD8^+^ T cells possess high proliferative capacity and can differentiate into transitional intermediate effector-like exhausted cells, characterized by CX3CR1 expression (Beltra et al., 2020; Giles et al., 2023; Paley et al., 2012). These intermediate cells subsequently progress into Tim3^*+*^ terminally exhausted cells. Furthermore, the expression of certain inhibitory receptors, such as PD-1 and LAG-3 (Johnnidis et al., 2021), initially declines slightly before substantially increasing during the exhaustion process (Giles et al., 2023).

Studies across multiple animal tumor models and patient samples have further revealed the heterogeneity of tumor-infiltrating exhausted CD8^+^ T cells (Baessler and Vignali, 2024; Huang et al., 2025). Tumor antigen-specific exhausted CD8^+^ T cells within the tumor microenvironment (TME) can be broadly categorized into three subsets: Tcf1^+^ Tim3^−^ PD-1^int^ progenitor exhausted T (T_PEX_) cells, Tcf1^−^ CX3CR1^+^ PD-1^hi^ intermediate exhausted T (Int-T_EX_) cells, and Tcf1^−^ CX3CR1^−^ Tim3^+^ PD-1^hi^ terminal exhausted T (T_EX_) cells (Brummelman et al., 2018; Hudson et al., 2019; Miller et al., 2019; Siddiqui et al., 2019; Zander et al., 2019). In cancer immunotherapy, reversing T cell exhaustion is critical. An important therapeutic objective is to promote the differentiation of T_EX_ cells into more effector-like Int-T_EX_ cells while preventing their progression into terminal T_EX_ cells, which are less functional. Identifying and understanding the critical factors that regulate this differentiation process remains a key focus for advancing effective immunotherapy strategies.

The exhaustion of CD8^+^ T cells is governed by a complex network of transcription factors. For example, tumor-infiltrating T_PEX_ cells rely on the transcription factor Tcf1 for early differentiation and maintenance. Tcf1 induces the expression of transcription factors such as Eomes, Bcl6, and c-Myb (Chen et al., 2019; Tsui et al., 2022; Wu et al., 2016), which promote the survival of T_PEX_ cells, enable self-renewal, and provide resistance to apoptosis. Furthermore, transcription factors, such as BACH2 and SATB1, also regulate the chromatin accessibility, transcriptional activity, and genome architecture of Tcf1 (Heyden et al., 2025; Lin et al., 2025; Yao et al., 2021a). Concurrently, Tcf1 antagonizes effector-related transcription factors, including Blimp1, ID2, RUNX3, and T-bet, stabilizing the stem-like characteristics of T_PEX_ cells (Chen et al., 2019; Shan et al., 2017; Sun et al., 2023; Utzschneider et al., 2020). As the exhaustion process progresses, Tcf1 expression is downregulated, and sustained antigen stimulation induces the upregulation of BATF/IRF4, T-bet, and IRF8 (Hudson et al., 2019; Li et al., 2025; Paley et al., 2012). T-bet further activates the transcription of Blimp-1 (Hwang et al., 2016) and ID2 (He et al., 2016), driving the transition of T_PEX_ into Int-T_EX_ cells, which secrete elevated levels of effector molecules (Beltra et al., 2020). Meanwhile, continuous TCR activation maintains the expression of NFAT, which ultimately induces the upregulation of the transcription factor Tox (Khan et al., 2019; Scott et al., 2019; Wang et al., 2019), leading to the differentiation of Int-T_EX_ cells into T_EX_ cells. Despite this understanding, how to effectively promote T_PEX_ cell differentiation toward a more functional state by modulating Tcf1 expression remains unclear. Addressing this challenge is critical for optimizing therapeutic strategies that aim to restore T cell function.

In recent years, epigenetic transcriptional regulation, particularly RNA modifications, has garnered significant attention. Among these, *N6*-methyladenosine (m^6^A) modification has been shown to play an important role in CD8^+^ T cell–mediated antitumor immunity (Wan et al., 2022). However, the potential contributions of other RNA modifications to CD8^+^ T cell function remain less well understood.

In our study, we identified *Mettl8*, an m^3^C methyltransferase, as being highly expressed in T_PEX_ cells. Using a conditional *Mettl8* deficiency model in mouse T cells, we demonstrated that *Mettl8* is critical for T_PEX_ cell maintenance. Loss of *Mettl8* resulted in the generation of more effective CD8^+^ T cells and significantly inhibited tumor growth. Mechanistically, Mettl8 was found to stabilize m^3^C-modified *Tcf7* mRNA, ensuring its functional persistence. Additionally, Mettl8 interacts with Tcf1 protein to co-bind genome-specific loops and regulate chromatin accessibility. These findings highlight the essential role of Mettl8 in regulating T_PEX_ cell stability and suggest its potential as a therapeutic target to enhance CD8^+^ T cell antitumor immunity.

## Results

### Mettl8 is highly expressed in stem-like T_PEX_ cells and reduced in response to anti–PD-1 therapy

**Figure 1.**
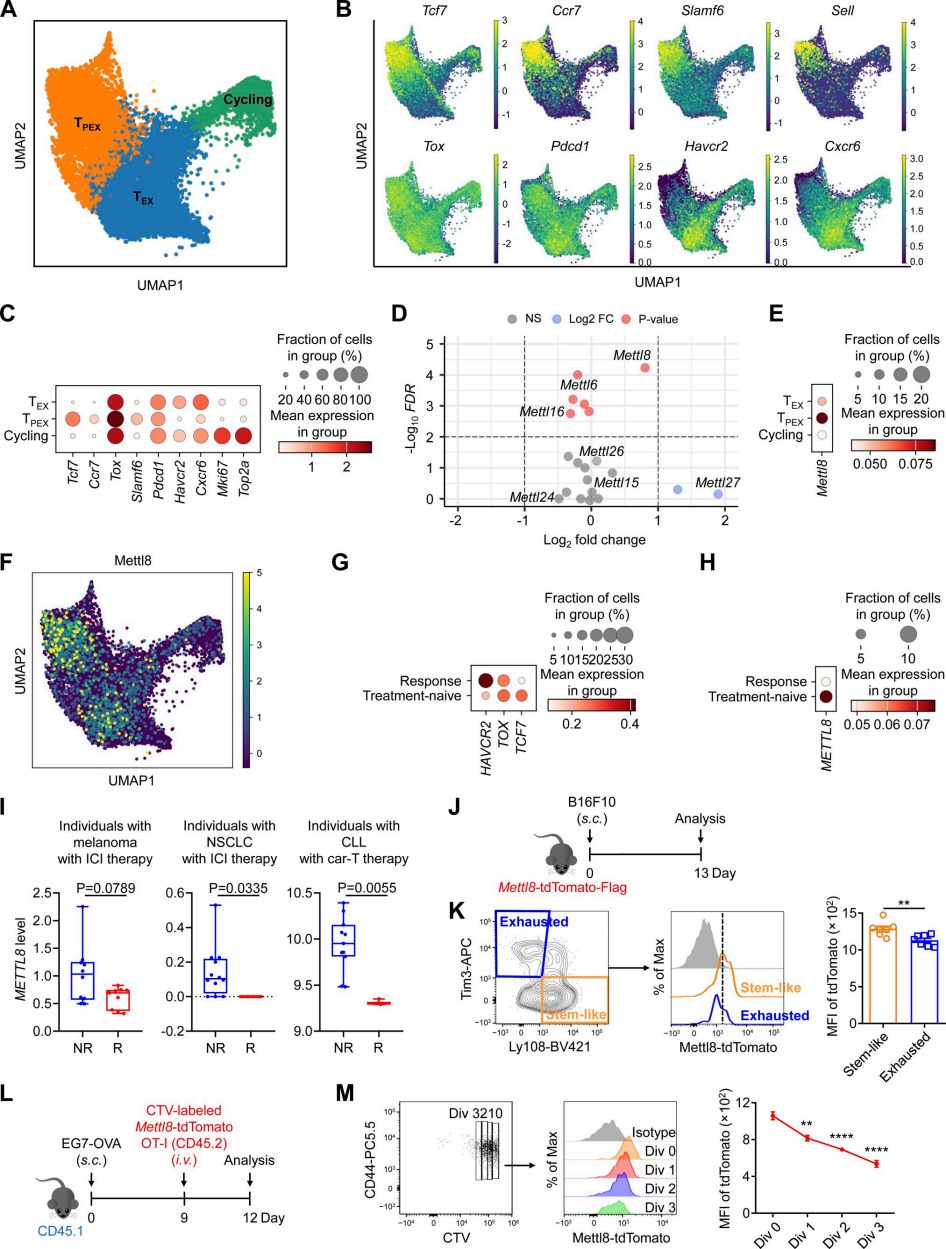
**Mettl8 is highly expressed in T**_**PEX**_ **cells and decreased after anti–PD-1 treatment. (A)** UMAP plot showing the clustering of OT-I cells infiltrating the MC38-OVA tumor. **(B and C)** UMAP (B) and bubble (C) plots showing the marker genes expression in each cluster. **(D)** Volcano plot displaying the fold change expression of *Mettl* genes in T_PEX_ cells compared with T_EX_ cells. Genes were considered significant if the adjusted P value (FDR) was <0.01. **(E and F)** Bubble (E) and UMAP (F) plots showing the expression of *Mettl8* in each cluster. **(G and H)** Bubble plots showing the expression of *HAVCR2*, *TOX*, *TCF7* (G), and *METTL8* (H) in the CD8^+^ T cells of cancer patients before (treatment-naïve) and after (response) anti–PD-1 treatment. **(I)** Relative expression of *METTL8* in tumor-infiltrating T cells from anti–PD-1/CTLA-4–treated melanoma (left) and anti-CD19 CAR-T–treated chronic lymphocytic leukemia (CLL) (right) responders (R) and nonresponders (NR) and in TILs from nivolumab treated NSCLC (middle) R and NR assessed by Tres (https://resilience.ccr.cancer.gov/). Each dot represents a tumor with the average value among all cells on the y axis. The thick line represents the median value. The bottom and top of the boxes are the min and max, respectively. (Melanoma: R = 9, NR = 10; NSCLC: R = 4, NR = 12; CLL: R = 3, NR = 11.) **(J)** Schematic diagram of the tumor model: Mettl8-tdTomato-Flag mice were subcutaneously injected with 2 × 10^5^ B16F10 cells. Mice were harvested at 13 dpi. **(K)** Representative cytometry plots showing the Ly108^+^ Tim3^−^ T_PEX_ and Tim3^+^ Ly108^−^ T_EX_ cells (left). Cells are gated on tumor-infiltrating CD8^+^ CD44^+^ T cells. Representative cytometry plots (middle) and statistic diagram (right) showing the expression of Mettl8-tdTomato in these cells. *n* = 6–7 per group. **(L)** Schematic diagram of the tumor model: CD45.1 mice were subcutaneously injected with 2 × 10^5^ EG7-OVA cells, followed by 2 × 10^6^ CTV-labeled Mettl8-tdTomato-Flag OT-I cells transfer at 9 dpi. Mice were harvested at 12 dpi. **(M)** Representative cytometry plots showing the divisions of CD44^+^ OT-I cells at day 3 after transfer (left). Representative cytometry plots (middle) and statistic diagram (right) showing the expression of Mettl8-tdTomato in each division. The mean fluorescence intensity (MFI) of tdTomato in division 1, 2, and 3 are all compared with that in division 0. *n* = 5 per group. Data are representative of two independent experiments. P values were calculated two-tailed Student’s *t* test, **P < 0.01; ****P < 0.0001.

**Figure S1.**
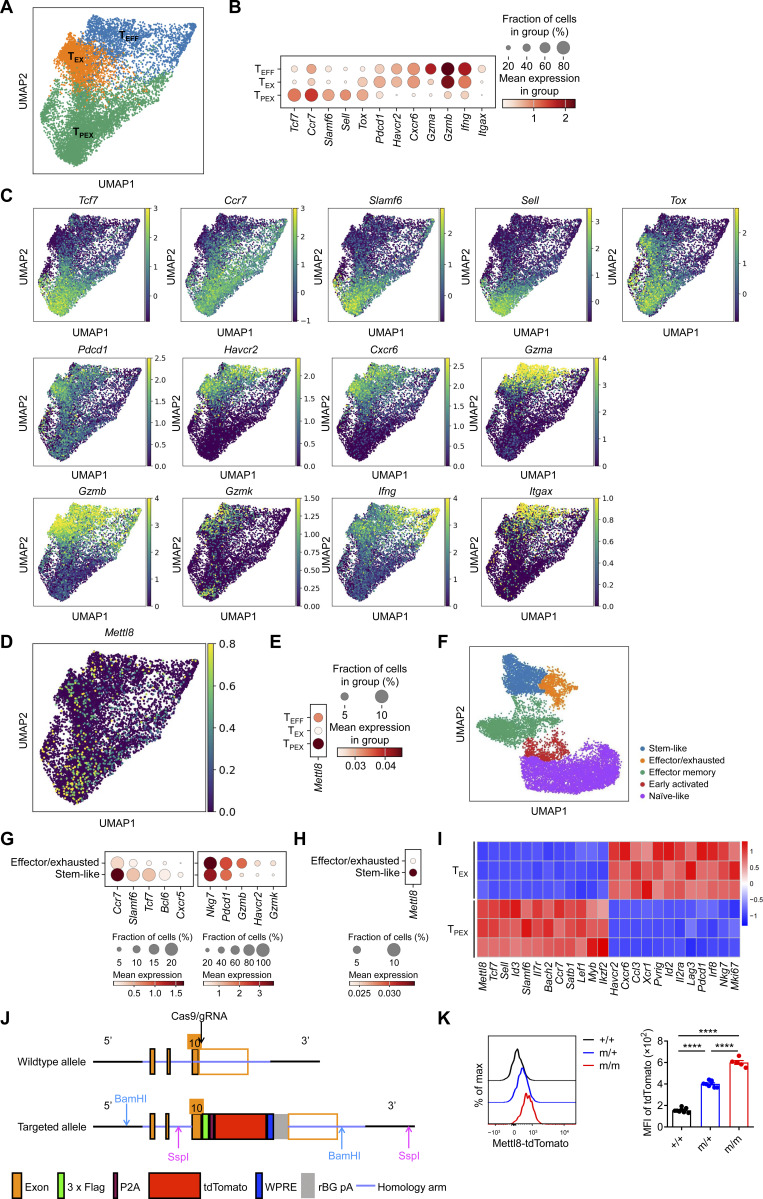
**Mettl8 expression in CD8** ^**+**^ **T cell subsets and generation of Mettl8-tdTomato-Flag mice. (A)** UMAP plot showing the clustering of 9,557 OT-I cells infiltrating in the B16-OVA tumor. **(B and C)** Bubble (B) and UMAP (C) plots showing the character gene expression in each cluster. **(D and E)** UMAP (D) and bubble (E) plots showing the expression of *Mettl8* in each cluster. **(F)** UMAP plot showing the clustering of CD8^+^ cells infiltrating in the mouse melanoma and colon adenocarcinoma tumor model. **(G and H)** Bubble plot showing signature gene (G) and *Mettl8* (H) expression in effector/exhausted and stem-like clusters. **(I)** RNA-seq analysis was performed on tumor-infiltrating OT-I cells sorted into Ly108^+^ Tim3^−^ T_PEX_ and Tim3^+^ Ly108^−^ T_EX_ populations. Heatmaps depict signature genes associated with T_PEX_ and T_EX_ cells (Log_2_FC > 1, FDR < 0.05). **(J)** Targeting strategy of *Mettl8* allele. **(K)** Expression of tdTomato in T cells of the spleens from Mettl8-tdTomato-Flag mice. *n* = 5–8 per group. Data are representative of two independent experiments. P value was calculated by one-way ANOVA; ****P < 0.0001.

To investigate the epigenetic regulators involved in the transition of T_PEX_ cells to T_EX_ cells, we reanalyzed single-cell RNA sequencing (scRNA-seq) data from 17,673 OT-I cells derived from MC38-OVA tumor models (Zhang et al., 2024) and 9,557 OT-I cells from B16-OVA model (Zhou et al., 2023). The analysis revealed three distinct clusters: T_PEX_, T_EX_, and cycling (T_EFF_) cells (Fig. 1 A and Fig. S1 A). As previously reported, stemness markers such as *Tcf7*, *Ccr7*, *Slamf6*, and *Sell* were highly expressed in T_PEX_ cells, whereas exhaustion markers, including *Pdcd1* and *Havcr2*, showed elevated expression in T_EX_ cells compared with T_PEX_ cells (Fig. 1, B and C; and Fig. S1, B and C). Since CXCR6 was reported to position Tcf1^−^ transitory CD8^+^ cytotoxic lymphocytes within the tumor stroma (Di Pilato et al., 2021), our analysis also revealed higher expression of *Cxcr6* in T_EX_ cells than that in T_PEX_ cells (Fig. 1, B and C; and Fig. S1, B and C). To identify RNA methyltransferases potentially contributing to the T_PEX_-to-T_EX_ transition, we compared the expression of RNA methyltransferase genes between these two cell populations. Among the *Mettl* gene family, *Mettl8* was significantly more highly expressed in T_PEX_ cells than in T_EX_ cells (Fig. 1 D). Further analysis confirmed this differential expression, showing consistently higher levels of *Mettl8* in T_PEX_ cells (Fig. 1, E and F; and Fig. S1, D and E). To further validate the expression of Mettl8 in CD8^+^ T cell subsets, we reanalyzed published scRNA-seq data from melanoma and colon adenocarcinoma samples (Andreatta et al., 2021). The data revealed several CD8^+^ T cell clusters, including a stem-like cluster that serves as the progenitor for effector/exhausted cells during the process of T cell exhaustion (Fig. S1 F). The stem-like cluster was characterized by high expression of canonical stemness markers such as *Tcf7*, *Slamf6*, *Bcl6*, *Cxcr5*, and *Ccr7* (Fig. S1 G). In contrast, expression of effector/exhausted markers, including granzyme B (*Gzmb*), *Gzmk*, and *Havcr2*, was reduced in the stem-like cluster compared with the effector/exhausted cluster (Fig. S1 G). Notably, *Mettl8* expression was significantly enriched in the stem-like cluster, aligning with the expression of other stemness-associated genes (Fig. S1 H). To further confirm the higher expression of Mettl8 in T_PEX_ compared with T_EX_ populations, we conducted bulk RNA-seq on sorted T_PEX_ and T_EX_ cells. The data showed significantly higher levels of Mettl8 in T_PEX_ cells, consistent with other T_PEX_ signature genes such as *Tcf7* and *Slamf6* (Fig. S1 I). These findings indicate that *Mettl8* is highly expressed in T_PEX_ cells, consistent with its association with stem-like signatures.

Tumor immune checkpoint inhibitor (ICI) therapy enhances the antitumor immunity of CD8^+^ T cells. To investigate the effect of Mettl8 during this process, we analyzed gene expression changes in CD8^+^ T cells from patients before and after anti–PD-1 treatment, utilizing the published scRNA-seq data of 59,586 cells from human non-small cell lung cancer (NSCLC) patients (Xue et al., 2024). Notably, following anti–PD-1 treatment, expression of the stemness gene *TCF7* was significantly downregulated, whereas the exhaustion marker *HAVCR2* was markedly upregulated (Fig. 1 G). Similarly, the expression of *METTL8* was also significantly decreased (Fig. 1 H), paralleling the reduction in *TCF7* expression. These findings suggest that anti–PD-1 therapy promotes the transition of T_PEX_ cells to T_EX_ cells, accompanied by a decline in *METTL8* expression.

Additionally, we analyzed potential associations between *METTL8* expression levels and the response to ICI or anti-CD19 CAR-T therapy using RNA-seq data (Xiang et al., 2025). Our results revealed that low *METTL8* expression in tumor-infiltrating T cells or tumor-infiltrating lymphocytes (TILs) was associated with an improved response to ICI or CAR-T therapy across various cancer types (Fig. 1 I). These findings suggest a negative correlation between *METTL8* expression and cancer immunotherapy efficacy, highlighting the potential inhibitory role of *METTL8* in T cell–mediated antitumor immunity.

To validate Mettl8 expression in T_PEX_ cells, we generated Mettl8-tdTomato-Flag mice (Fig. S1 J). Analysis of CD8^+^ T cells confirmed that tdTomato signal was indeed strongest in homozygous cells, whereas heterozygous and WT cells showed lower fluorescence (Fig. S1 K). These data verify the successful generation of the reporter line. We then examined CD8^+^ T cells within the TME of Mettl8-tdTomato-Flag mice (Fig. 1 J). Notably, Mettl8 expression was significantly higher in Ly108^+^ Tim3^−^ stem-like cells compared with Tim3^+^ Ly108^−^ exhausted cells (Fig. 1 K). Since the early differentiation of tumor-specific CD8^+^ T cells in tumor-draining LNs (TdLNs) is critical for their response to PD-1/PD-L1 blockade (Huang et al., 2022), we crossed Mettl8-tdTomato-Flag mice with OT-I mice. We then transferred CellTrace Violet (CTV)-labeled Mettl8-tdTomato-Flag OT-I cells into EG7-OVA tumor-bearing mice (Fig. 1 L). Analysis of these cells 3 days after transfer showed a gradual decrease in Mettl8 expression as the cells underwent division (Fig. 1 M). These findings indicate that Mettl8 expression declines progressively during the development of CD8^+^ T cells, aligning with the transition from T_PEX_ to T_EX_ cells. In summary, Mettl8 is highly expressed in stem-like T_PEX_ cells and diminishes during cell development and in response to anti–PD-1 therapy.

### Mettl8 suppresses T_PEX_ cell differentiation to T_EX_ cells

**Figure S2.**
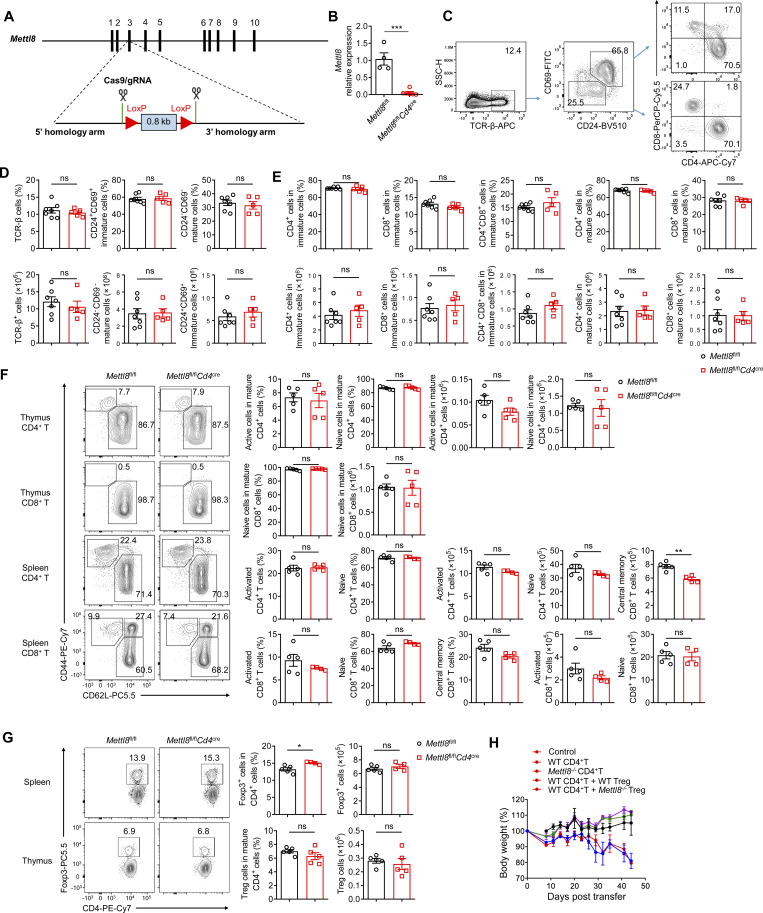
**T cell maturation in the thymuses and spleens of Mettl8 conditional KO mice. (A)** Schematic diagram of “conditional ready” (floxed) *Mettl8* allele. Two loxP sites (red triangle) flanking the *Mettl8* exon (blue square) within the 5′ and -3′ homology regions (∼1.5 kb, respectively) are indicated. **(B)** Relative expression of *Mettl8* in CD3^+^ T cells from the spleens of *Mettl8*^fl/fl^ (WT) and *Mettl8*^fl/fl^*Cd4*^cre^ (KO) mice, detected by qPCR. **(C−E)** Representative plots (C) and cumulative data (D and E) show different states of T cell development in the thymus of WT and KO mice as tested by flow cytometry. **(F)** Representative flow cytometry plots and cumulative data show the frequency and absolute number of CD44^hi^ CD62L^lo^ effector memory cells and CD44^lo^ CD62L^hi^ naïve cells gated on TCRβ^+^ CD24^−^ CD69^−^ mature CD4^+^ T cells (top) and mature CD8^+^ T cells (upper middle) from the thymuses, CD44^hi^ CD62L^lo^ active cells, CD44^lo^ CD62L^hi^ naïve cells, and CD44^hi^ CD62L^hi^ central memory cells gated on CD4^+^ T cells (lower middle) and mature CD8^+^ T cells (bottom) from the spleens of *Mettl8*^fl/fl^*Cd4*^cre^ mice and littermate controls. **(G)** Representative flow cytometry plots and cumulative data show the frequency and absolute number of Foxp3^+^ CD4^+^ T cells the spleens (top) and the thymuses (bottom) of *Mettl8*^fl/fl^*Cd4*^cre^ mice and littermate controls. **(H)** Body weight of enteritis mice induced by transfer of CD4^+^ T cell to *Rag1*^−/−^ mice and suppression by *Mettl8*^−/−^ or WT T_reg_ cells. *n* = 4–7 mice per group. Data are representative of two independent experiments. P value was calculated by two-tailed Student’s *t* test. P value was calculated by two-tailed Student’s *t* test; *P < 0.05; **P < 0.01; ***P < 0.001.

**Figure 2.**
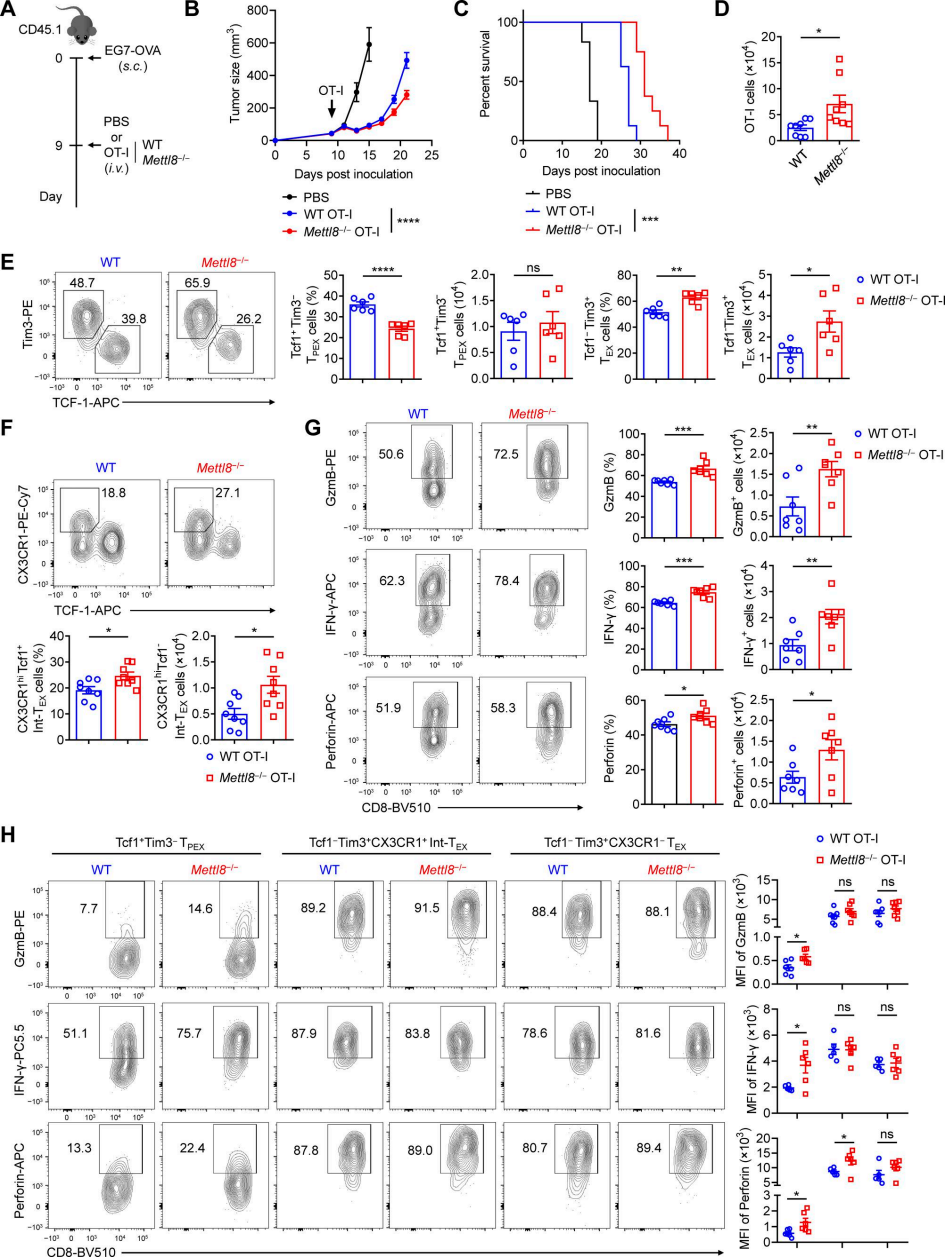
***Mettl8* deficiency restricts tumor progression by promoting T**_**PEX**_ **cell transition. (A)** Schematic diagram of the adoptive transferred tumor model: CD45.1 mice were subcutaneously injected with 2 × 10^5^ EG7-OVA cells, followed by 2 × 10^6^ CD45.2 WT or *Mettl8*^−/−^ OT-I cells transfer at 9 dpi. Mice were harvested at 21 dpi. **(B)** Tumor growth in each group of the mice in A. *n* = 8 per group. **(C)** Survival curve in each group of the mice in A. *n* = 8 per group. **(D)** The absolute number of tumor infiltrating OT-I cells from the mice in A. *n* = 8 per group. **(E and F)** Representative flow cytometry plots and cumulative data show the frequency and absolute number of Tcf1^+^ Tim3^−^ T_PEX_, Tim3^+^ Tcf1^−^ T_EX_ (E), and CX3CR1^+^ Tcf1^−^ Int-T_EX_ cells (F) gated on tumor-infiltrating OT-I cells. *n* = 6–8 per group. **(G)** Representative flow cytometry plots (left) and cumulative data (right) show the frequency and absolute number of GzmB^+^, IFN-γ^+^, and perforin^+^ cells gated on tumor-infiltrating OT-I cells. *n* = 7 per group. **(H)** Representative flow cytometry plots (left) and cumulative data (right) show the frequency and MFI of GzmB, IFN-γ, and perforin gated on tumor-infiltrating Tcf1^+^ Tim3^−^ T_PEX_, CX3CR1^+^ Tcf1^−^ Int-T_EX,_ and CX3CR1^−^ Tcf1^−^ T_EX_ subsets. *n* = 5–6 per group. Data are representative of three independent experiments. P value was calculated by two-way ANOVA (B), Log-rank test (C), and two-tailed Student’s *t* test (D−H); *P < 0.05; **P < 0.01; ***P < 0.001; ****P < 0.0001.

**Figure S3.**
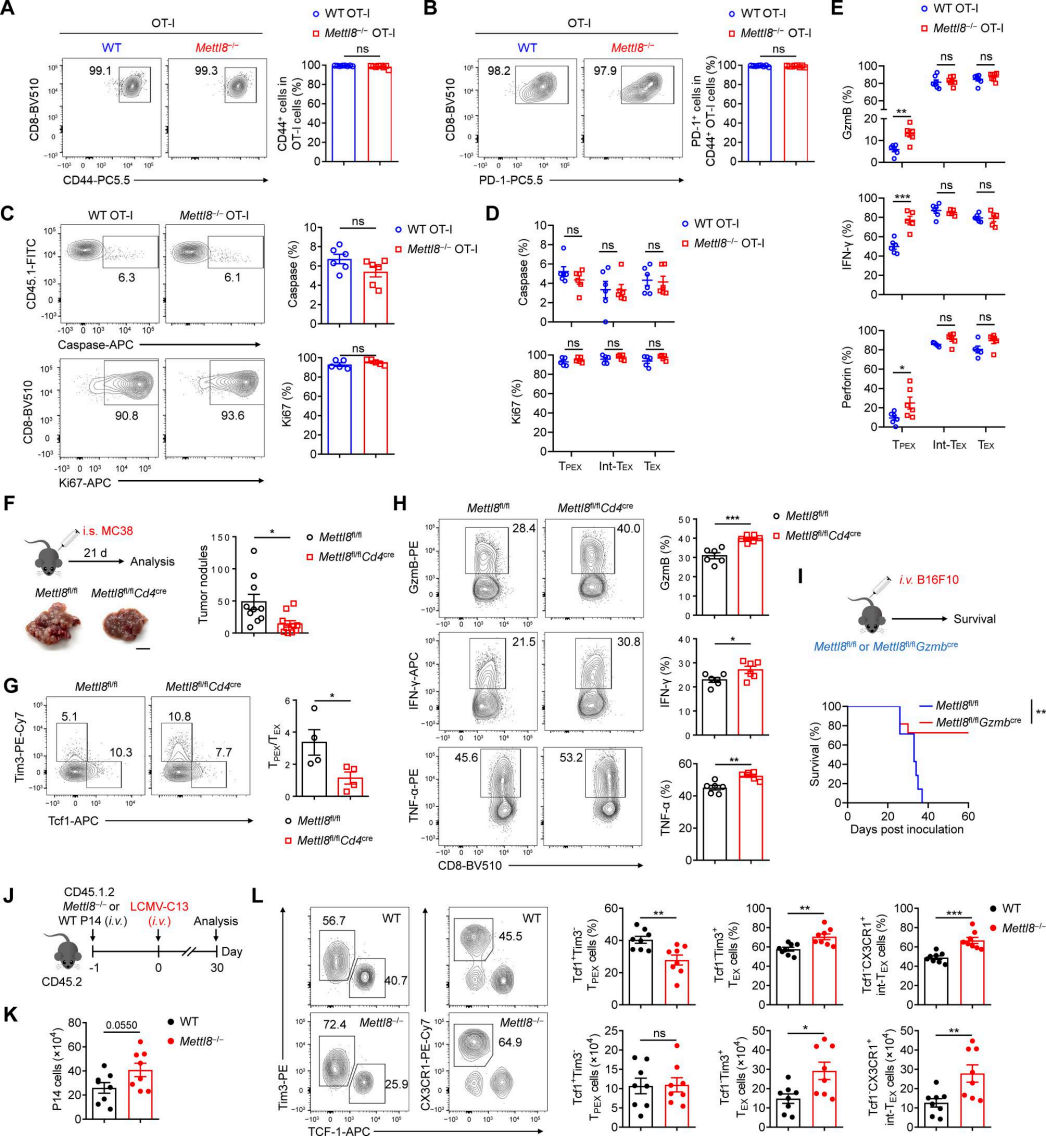
**Mettl8 promotes T**_**PEX**_ **differentiation without affecting their proliferation and apoptosis. (A and B)** Representative flow cytometry plots and cumulative data show the frequency of CD44 (A) and PD-1 (B) OT-I cells infiltrating in tumors. **(C)** Representative flow cytometry plots and cumulative data show the frequency of caspase and Ki67 in tumor-infiltrating OT-I cells. **(D)** Cumulative data show the frequency of caspase and Ki67 in Tcf1^+^ Tim3^−^ T_PEX_, CX3CR1^+^ Tcf1^−^ Int-T_EX_, and CX3CR1^−^ Tcf1^−^ T_EX_ subsets. **(E)** Cumulative data show the frequency of GzmB, IFN-γ, and perforin in tumor-infiltrating OT-I subsets mentioned above. **(F)** Schematic diagram of the classic CRC liver metastases model: *Mettl8*^fl/fl^*Cd4*^*cre*^ and *Mettl8*^fl/fl^ mice were intrasplenically injected with 2 × 10^5^ MC38 cells (left), and imaging of livers on day 21 after injection (right). **(G)** Representative flow cytometry plots and cumulative data show the ratio of Tcf1^+^ Tim3^−^ T_PEX_ to Tim3^+^ Tcf1^−^ T_EX_ cells gated on CD44^hi^ CD62L^lo^ CD8^+^ T cells of the livers from mice in F. **(H)** Representative flow cytometry plots and cumulative data show the frequency of GzmB, IFN-γ, and TNF-α gated on CD44^hi^ CD62L^lo^ CD8^+^ T cells from the livers of mice in F. **(I)** Schematic diagram of the classic melanoma lung metastases model: *Mettl8*^fl/fl^*Gzmb*^cre^ and *Mettl8*^fl/fl^ mice were i.v. injected with 2 × 10^5^ B16F10 cells (top) and the survival curve (bottom). **(J)** Schematic diagram of adoptive transfer model: CD45.1.2^+^*Mettl8*^−/−^ or WT P14 CD8^+^ T cells were adoptively transferred into CD45.2^+^ WT recipients, followed by LCMV-clone 13 (LCMV-C13) infection 24 h later and then analyzed on 30 dpi. **(K)** Statistical analysis show the absolute number of P14 cells from the spleens of mice in J. **(L)** Representative flow cytometry plots and cumulative data show Tcf1^+^ Tim3^−^ T_PEX_, Tim3^+^ Tcf1^−^ T_EX_, and CX3CR1^+^ Tcf1^−^ Int-T_EX_ cells gated on P14 cells from the spleens of mice in J. *n* = 4–8 mice per group. Data are representative of two independent experiments. P value was calculated by two-tailed Student’s *t* test (A–H, K, and L) or Log-rank test (I); *P < 0.05; **P < 0.01; ***P < 0.001.

To investigate the effect of Mettl8 in CD8^+^ T cells, we constructed Mettl8-flox mice and used CD4-cre to ablate Mettl8 in αβ thymocytes (Fig. S2, A and B). T cell development in the thymus (Fig. S2, C–E) and the maturation of T cells in thymus and spleen were not affected by *Mettl8* deficiency (Fig. S2 F). Furthermore, *Mettl8*^fl/fl^*Cd4*^cre^ mice exhibited normal development and immunosuppressive function of regulatory T (T_reg_) cells (Xing et al., 2019) (Fig. S2, G and H). To assess the impact of Mettl8 on CD8^+^ T cell exhaustion, we crossed *Mettl8*^fl/fl^*Cd4*^cre^ mice with OT-I mice and transferred WT or *Mettl8*^−/−^ OT-I cells into mice subcutaneously injected with EG7-OVA tumors (Fig. 2 A). Mice receiving *Mettl8*^−/−^ OT-I cells exhibited significantly slower tumor growth and prolonged survival compared with those receiving WT OT-I cells (Fig. 2, B and C). Analysis of TILs showed a marked increase in the number of OT-I cells upon *Mettl8* deficiency (Fig. 2 D), without alterations in the frequency of CD44 and PD-1 expression (Fig. S3, A and B). Notably, *Mettl8* deficiency skewed OT-I cells toward a more effector/exhausted phenotype, as evidenced by a reduced proportion of Tcf1^+^ Tim3^−^ T_PEX_ cells, along with an increased proportion and number of CX3CR1^+^ Tcf1^−^ Int-T_EX_ and Tim3^+^ Tcf1^−^ T_EX_ cells, despite no change in the absolute number of T_PEX_ cells (Fig. 2, E and F). Moreover, neither apoptosis nor proliferation of OT-I cells or their subsets were affected by *Mettl8* deficiency (Fig. S3, C and D). Together, these results suggest that *Mettl8* deficiency promotes the differentiation of T_PEX_ cells to Int-T_EX_ and T_EX_ cells.

To evaluate the effector function of *Mettl8*-deficient OT-I cells, we tested the expression of key effector molecules, including GzmB, IFN-γ, and perforin. We found that the percentage of cytokine-producing cells as well as their absolute numbers were markedly elevated in *Mettl8*^−/−^ OT-I cells relative to WT cells. (Fig. 2 G). Given that different T cell subsets have varying capacities to express effector molecules, we further analyzed their expression within distinct exhausted subsets. Although T_PEX_ cells generally expressed the lowest levels among all subsets, *Mettl8* deficiency significantly upregulated these effector molecules specifically within the T_PEX_ population. In contrast, the expression in Int-T_EX_ and T_EX_ cells was unaffected (Fig. 2 H and Fig. S3 E). This subset-specific effect is consistent with the model that Mettl8 deficiency promotes the differentiation of T_PEX_ cells into more effector-competent downstream subsets. Collectively, these results suggest that Mettl8 helps maintain tumor-specific CD8^+^ T cells in a less differentiated, stem-like state within the TME.

To investigate the generality of Mettl8’s role in CD8^+^ T cells, we employed multiple *in vivo* models. In a liver metastasis model established by intrasplenic injection of MC38 cells, *Mettl8*^fl/fl^*Cd4*^cre^ mice developed significantly fewer tumor nodules than littermate controls (Fig. S3 F). This was accompanied by a pronounced decrease in the ratio of Tcf1^+^ Tim3^−^ T_PEX_ cells to Tim3^+^ Tcf1^−^ T_EX_ cells (Fig. S3 G), alongside significantly elevated proportion of GzmB, IFN-γ, and TNF-α (Fig. S3 H). To further validate the activated T cell–intrinsic effect, we crossed *Mettl8*^fl/fl^ mice with *Gzmb*^cre^ mice and i.v. injected B16F10 melanoma cells. *Mettl8* deficiency conferred a significant survival advantage in this lung metastasis model (Fig. S3 I). Finally, using an LCMV-clone13 chronic infection model with adoptive transfer of *Mettl8*^−/−^ P14 cells (Fig. S3 J), we observed an increasing trend of P14 cell numbers (Fig. S3 K). We also found a reduced proportion of T_PEX_ cells alongside expanded Int-T_EX_ and T_EX_ cell populations (Fig. S3 L), a phenotype consistent with our findings in tumor models. Collectively, these results from diverse models demonstrate that Mettl8 acts as a broad suppressor of the transition from the stem-like T_PEX_ cells to effector-like Int-T_EX_ cells and T_EX_ cells in CD8^+^ T cells.

**Figure 3.**
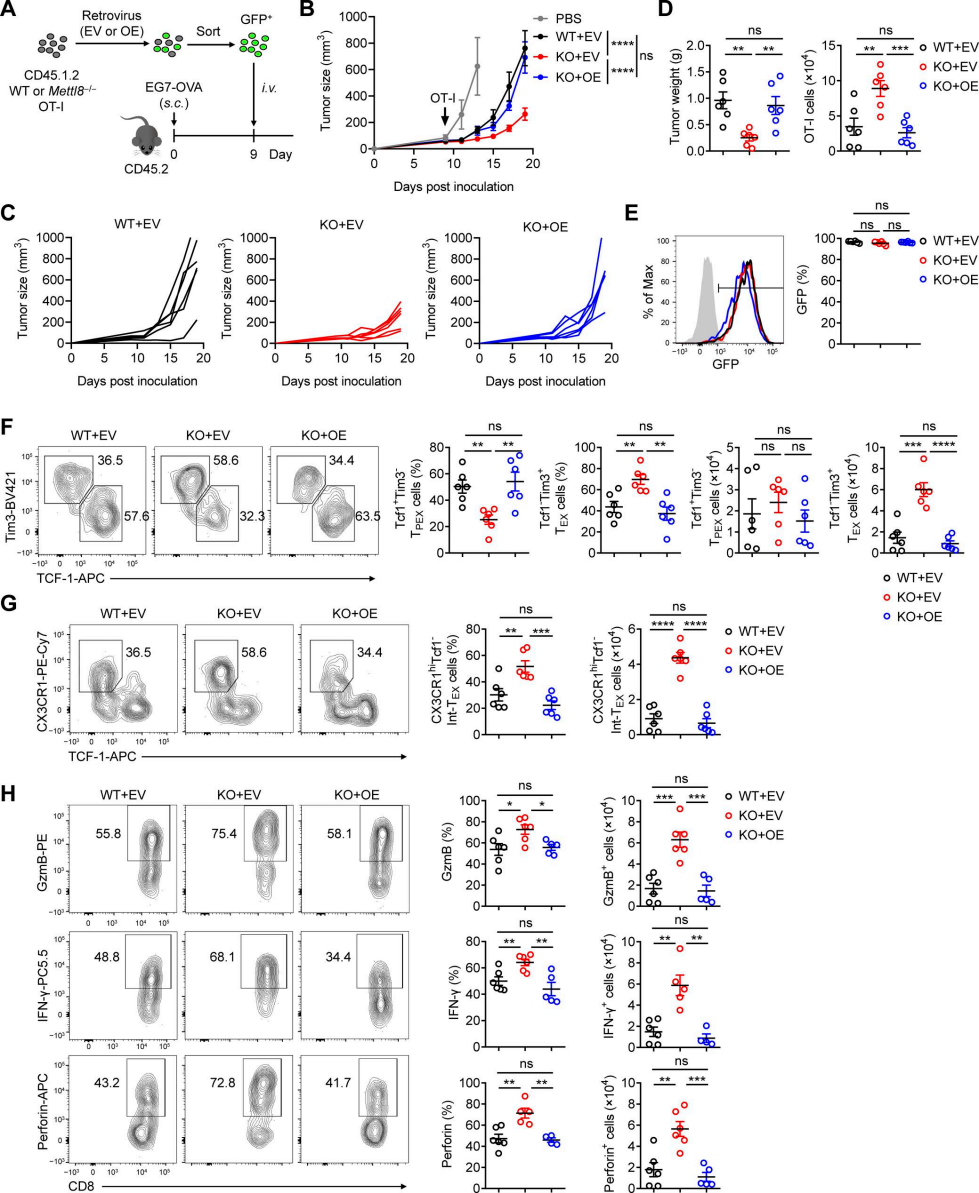
**Reconstitution of Mettl8 expression in *Mettl8*** ^**−/−**^ **OT-I cells restored their phenotype to that of WT OT-I cells. (A)** Schematic diagram of the rescue experiment: CD45.2 mice were subcutaneously injected with 2 × 10^5^ EG7-OVA cells. Mettl8 overexpression (OE) or empty vector (EV) retrovirus were transduced to CD45.1.2 WT or *Mettl8*^−/−^ OT-I cells. 5 × 10^5^ GFP^+^ cells were sorted 48 h after transduction and adoptively transferred into the tumor-bearing mice at 9 dpi. Mice were harvested at 19 dpi. **(B)** Tumor growth in each group of the mice in A. *n* = 6 per group. **(C)** Tumor growth in each group displayed in each replicate. *n* = 6 per group. **(D)** Tumor weight (left) and the absolute number of tumor infiltrating OT-I cells (right) from the mice in A. *n* = 6 per group. **(E)** Representative flow cytometry plots and cumulative data show the frequency of GFP in OT-I cells. *n* = 6 per group. **(F)** Representative flow cytometry plots (left) and cumulative data (right) show the frequency and absolute number of Tcf1^+^ Tim3^−^ T_PEX_ and Tim3^+^ Tcf1^−^ T_EX_ cells gated on tumor-infiltrating OT-I cells. *n* = 6 per group. **(G)** Representative flow cytometry plots (left) and cumulative data (right) show the frequency and absolute number of CX3CR1^+^ Tcf1^−^ Int-T_EX_ cells gated on tumor-infiltrating OT-I cells. *n* = 6 per group. **(H)** Representative flow cytometry plots (left) and cumulative data (right) show the frequency and absolute number of GzmB^+^, IFN-γ^+^, and perforin^+^ cells gated on tumor-infiltrating OT-I cells. *n* = 6 per group. Data are representative of two independent experiments. P value was calculated by two-way ANOVA (B) and two-tailed Student’s *t* test (D to H); *P < 0.05; **P < 0.01; ***P < 0.001; ****P < 0.0001.

To further validate that M*ettl8* deficiency promotes the T_PEX_-to-Int-T_EX_ transition and to rule out potential off-target effects from the constitutive KO model, we performed a rescue experiment by reconstituting Mettl8 expression in *Mettl8*^−/−^ OT-I cells using a pMSCV-IRES-GFP retrovirus (Fig. 3 A). Compared with the empty vector control, enforced Mettl8 expression in *Mettl8*^−/−^ OT-I cells impaired tumor control to a level comparable with that observed in WT OT-I cells transduced with the empty vector (Fig. 3, B–D). Analysis of tumor-infiltrating OT-I cells revealed high GFP positivity, confirming successful transduction (Fig. 3 E). Subsequent examination of these GFP^+^ cells showed that the subset distribution and effector molecule proportion in *Mettl8*^−/−^ OT-I cells reconstituted with Mettl8 were similar to those in empty vector–transduced WT OT-I cells (Fig. 3, F–H). These rescue data firmly establish Mettl8 as a suppressor of T_PEX_ cell differentiation.

### Mettl8 promotes CD8^+^ T cell maintenance of the stem-like phenotype

**Figure 4.**
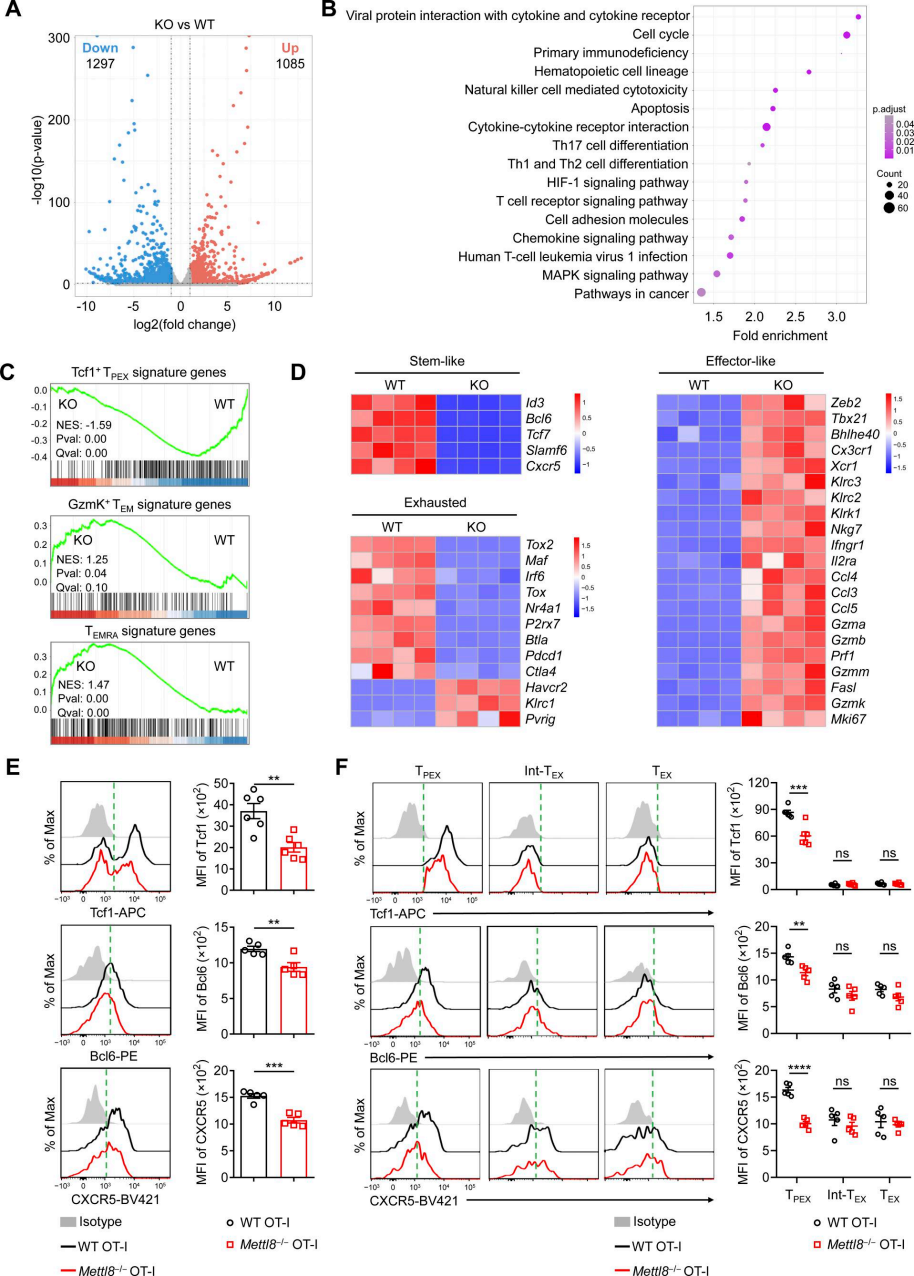
***Mettl8* deficiency alters transcriptional profiles of CD8** ^**+**^ **T cells.** RNA-seq analysis were performed using WT or *Mettl8*^−/−^ (KO) OT-I cells from the adoptive transferred tumor model. **(A)** Volcano plot illustrates genes differentially expressed between OT-I cells from WT and KO mice. Lines indicate a twofold difference between OT-I cells from KO and WT mice (DESeq2, FDR < 0.05). **(B)** Gene Ontology (GO) enrichment (biological processes) analysis of DEGs obtained from the R package named clusterProfiler. **(C)** GSEA shows enrichment of Tcf1^+^ T_PEX_ signature genes (top), GzmK^+^ T_EM_ signature genes (middle), and T_EMRA_ signature genes (bottom) in the transcriptome of KO versus WT cells. **(D)** Heatmaps of DEGs related to stem-like, exhausted, or effector-like features (Log2FC > 1, FDR < 0.05). **(E)** Representative flow cytometry plots (left) and cumulative data (right) show the expression of the indicated molecules gated on tumor-infiltrating OT-I cells. *n* = 5–6 per group. **(F)** Representative flow cytometry plots (left) and cumulative data (right) show the expression of the indicated molecules gated on tumor-infiltrating Tcf1^+^ Tim3^−^ T_PEX_, CX3CR1^+^ Tcf1^−^ Int-T_EX_, and CX3CR1^−^ Tcf1^−^ T_EX_ subsets. *n* = 5–6 per group. Data are representative of two independent experiments. P values were calculated by two-tailed Student’s *t* test; **P < 0.01; ***P < 0.001; ****P < 0.0001.

**Figure S4.**
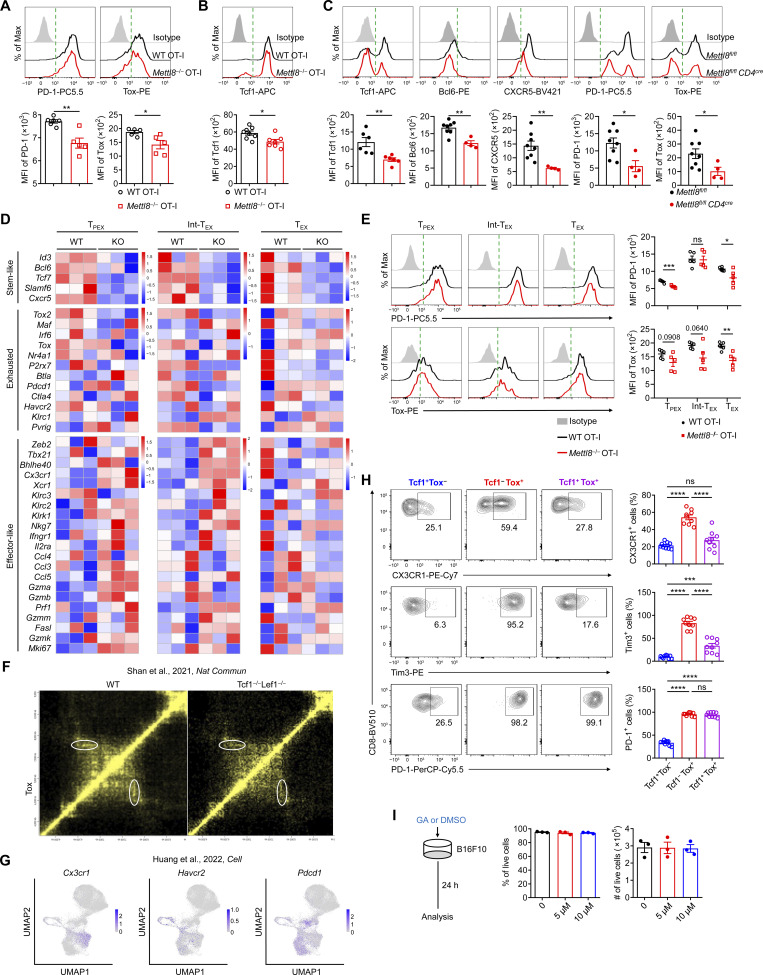
**The expression of effector/exhausted molecules across CD8** ^**+**^ **T cell subsets. (A)** Representative plots and cumulative data show the expression of PD-1 and Tox gated on tumor-infiltrating OT-I cells. *n* = 5 per group. **(B)** Representative plots and cumulative data show the expression of Tcf1 gated on OT-I cells in TdLNs. *n* = 4–8 per group. **(C)** Representative flow cytometry plots and cumulative data show the expression of the indicated molecules gated on tumor-infiltrating CD44^+^ CD8^+^ T cells in the B16F10 tumor model: *Mettl8*^fl/fl^ CD4^cre^ mice and littermate controls were subcutaneously injected with 2 × 10^5^ B16F10 cells. Mice were analyzed at 13 dpi. **(D)** RNA-seq analysis of Ly108^+^ Tim3^−^ T_PEX_, Tim3^+^ CX3CR1^+^ Ly108^−^ Int-T_EX_, and Tim3^+^ CX3CR1^−^ Ly108^−^ T_EX_ cells gated on tumor-infiltrating WT and Mettl8^−/−^ (KO) OT-I cells. Heatmaps depict stem-like, exhausted and effector-like gene signatures in WT and *Mettl8*^−/−^ (KO) OT-I cells. **(E)** Representative flow cytometry plots and cumulative data show the expression of PD-1 and Tox gated on tumor-infiltrating Tcf1^+^ Tim3^−^ T_PEX_, CX3CR1^+^ Tcf1^−^ Int-T_EX_, and CX3CR1^−^ Tcf1^−^ T_EX_ subsets. *n* = 5 per group. **(F)** Diamond graphs show chromatin interactions in WT (left) and Tcf1^−/−^ Lef1^−/−^ dKO (right) CD8^+^ T cells, with gene structures on the left. **(G)** Single-cell transcription levels of representative genes illustrated in the UMAP plot. Transcription levels are color coded: gray, not expressed; blue, expressed. **(H)** Representative flow and cumulative data show the frequencies of CX3CR1, Tim3, and PD-1 in Tcf1^+^ Tox^−^, Tcf1^−^ Tox^+^, and Tcf1^+^ Tox^+^ cells, which are gated from B16F10 tumor-infiltrating CD8^+^ CD44^+^ T cells. *n* = 9 per group. **(I)** Schematic diagram of GA treatment to B16F10 cells *in vitro* (left) and frequency and absolute number of GA-treated cells (right). *n* = 3 per group. Data are representative of two independent experiments. P value was calculated by two-tailed Student’s *t* test; *P < 0.05; **P < 0.01; ***P < 0.001; ****P < 0.0001.

To comprehensively understand the impact of *Mettl8* deficiency on CD8^+^ T cells, we conducted RNA-seq on sorted tumor-infiltrating CD8^+^ T cells. This analysis identified 1,297 downregulated genes and 1,085 upregulated genes by *Mettl8* deficiency (Fig. 4 A). These differentially expressed genes (DEGs) were notably enriched in pathways related to cell cytotoxicity and pathways in cancer (Fig. 4 B). Gene set enrichment analysis (GSEA) further demonstrated that Mettl8-deficient CD8^+^ T cells exhibited a reduced enrichment in Tcf1^+^ T_PEX_ signature genes compared with WT cells (Zheng et al., 2021), whereas gene sets associated with effector phenotypes, including GzmK^+^ Tem and Temra signature genes (Zheng et al., 2021), were enriched in the absence of *Mettl8* (Fig. 4 C). Notably, *Tcf7*, which encodes Tcf1, was downregulated in *Mettl8*-deficient CD8^+^ T cells, while transcription factors linked to T cell activation and exhaustion, such as *Tbx21*, *Bhlhe40*, and *Zeb2*, were upregulated (Fig. 4 D). *Mettl8* deficiency also elevated the expression of inhibitory receptors, including Tim3 (*Havcr2*), NKG2A (*Klrc1*), and PVRIG (Fig. 4 D). Additionally, genes encoding cytolytic granules and other effector molecules, such as *Gzma*, *Gzmb*, *Prf1*, and *Fasl*, were expressed at higher levels in *Mettl8*-deficient CD8^+^ T cells compared with WT controls (Fig. 4 D). Furthermore, flow cytometric analysis confirmed a significant reduction in the expression of “stem-like” characters Tcf1, Bcl6, and CXCR5, as well as “exhausted” characters PD-1 and Tox in *Mettl8*-deficient CD8^+^ T cells (Fig. 4 E and Fig. S4 A). Given that CD8^+^ T cells in the LNs are predominant Tcf1^+^ T_PEX_ cells (Connolly et al., 2021; Huang et al., 2022; Prokhnevska et al., 2023), we also assessed Tcf1 expression of OT-I cells from TdLNs. *Mettl8* deficiency similarly reduced Tcf1 expression in these cells, mirroring the trend observed in intratumoral T_PEX_ cells (Fig. S4 B). We further validated these findings using a B16F10 melanoma model on *Mettl8*^fl/fl^*Cd4*^cre^ mice and littermate controls. The loss of Mettl8 induced similar alterations in stem-like and exhaustion-associated phenotypes in tumor-infiltrating CD8^+^ T cells, consistent with the observations in OT-I cells (Fig. S4 C). Collectively, these results indicate that *Mettl8*-deficient CD8^+^ T cells acquire genetic and phenotypic features indicative of an effector/exhausted lineage. Thus, *Mettl8* deficiency promotes CD8^+^ T cell effector function by driving a transition from a “stem-like” state toward an “effector/exhausted” state.

Considering the heterogeneous expression of these markers across T cell subsets, we further performed RNA-seq on sorted Ly108^+^ Tim3^−^ T_PEX_, Tim3^+^ CX3CR1^+^ Ly108^−^ Int-T_EX_, and Tim3^+^ CX3CR1^−^ Ly108^−^ T_EX_ subsets. Analysis revealed consistent downregulation of stem-like genes across all subsets upon *Mettl8* deficiency. However, genes associated with exhaustion or effector-like states remained largely unchanged (Fig. S4 D). At the protein level, expression of Tcf1, Bcl6, and CXCR5 was significantly reduced in T_PEX_ cells, while no significant changes were observed in the other two subsets (Fig. 4 F). This discrepancy is likely attributable to the inherently low baseline expression of these proteins in the Int-T_EX_ and T_EX_ subsets, making further reduction difficult to detect. PD-1 expression was significantly decreased in T_PEX_ and T_EX_ cells, but unchanged in Int-T_EX_ cells (Fig. S4 E). Tox showed a decreasing trend across all three subsets upon *Mettl8* deficiency (Fig. S4 E). Collectively, these results demonstrate that *Mettl8* deficiency selectively impairs the transcriptional program in T_PEX_ cells. Moreover, the overall changes observed in the bulk CD8^+^ T cell population are likely attributable to a reduced T_PEX_/T_EX_ ratio.

### Mettl8 regulates Tox expression via cooperation with Tcf1

**Figure 5.**
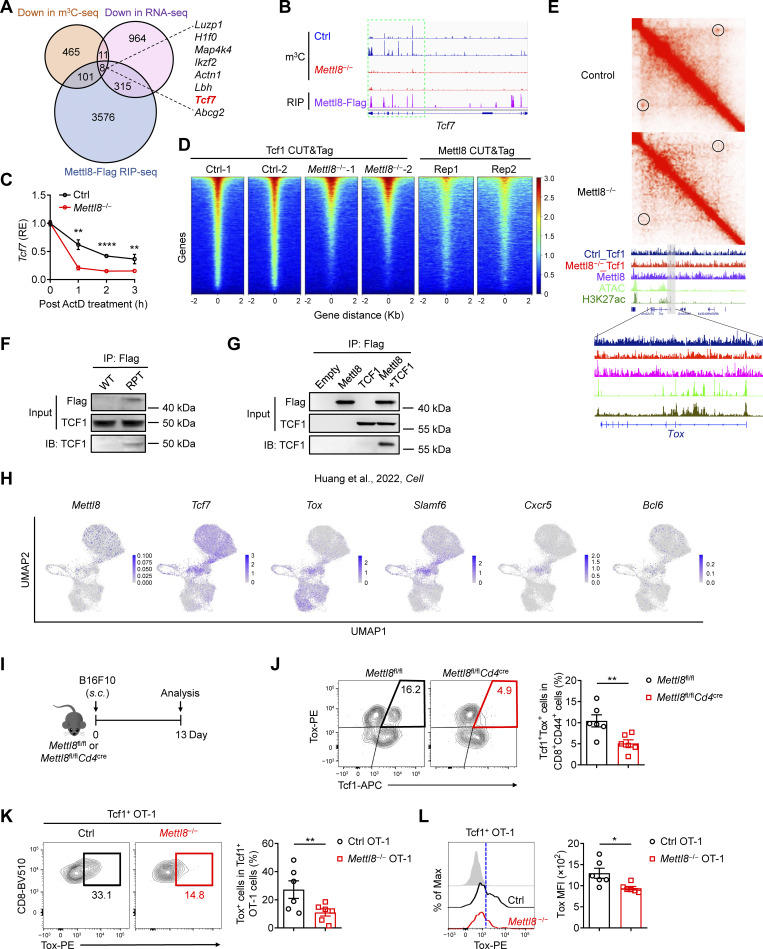
**Mettl8 promotes m** ^**3**^ **C modification of *Tcf7* mRNA and its genome-specific loops of Tox in CD8** ^**+**^ **T cells. (A)** Venn plot illustrates the overlap of downregulated genes from RNA-seq, m^3^C-seq, and Mettl8-binding genes from RIP-seq. **(B)** Mettl8 occupancy at the *Tcf7* gene loci is revealed through m^3^C-seq (WT and *Mettl8*^−/−^) of EG7-OVA tumor-infiltrating OT-I cells and RIP-seq (Mettl8-tdTomato-Flag) of B16F10 tumor-infiltrating CD44^+^ CD8^+^ T cells. The binding peaks on *Tcf7* loci are depicted. The m^3^C tracks are all plotted on a consistent scale. **(C)** The RNA decay assay demonstrates the remaining *Tcf7* mRNA of CD8^+^ T cells from the spleens of WT and *Mettl8*^−/−^ mice detected by qRT-PCR, normalized to t = 0. **(D)** Heatmaps display changes in total Tcf1-targeting genes between WT and *Mettl8*^−/−^ EG7-OVA tumor-infiltrating OT-I cells and Mettl8-targeting genes in B16F10 tumor-infiltrating CD44^+^ CD8^+^ T cells of Mettl8-tdTomato-Flag mice as detected by CUT&Tag. **(E)** Diamond graphs exhibit chromatin interactions in WT and *Mettl8*^−/−^ tumor-infiltrating OT-I cells at the *Tox* gene loci (top), with CUT&Tag and ATAC-seq tracks, and gene structures on the bottom. An enlarged view highlights the signal profiles across the *Tox* gene region. **(F)** co-IP of Tcf1 by anti-Flag magnetic beads in CD3^+^ T cells from the spleens of Mettl8-tdTomato-Flag (RPT) and WT mice. IB, immunoblot. **(G)** co-IP of Tcf1 by Flag-tagged Mettl8 protein with anti-Flag magnetic beads after co-transfection into HEK293T cells. **(H)** Single-cell transcription levels of representative genes illustrated in the UMAP plot. Transcription levels are color coded: gray, not expressed; blue, expressed. **(I)** Schematic diagram of the tumor model: *Mettl8*^fl/fl^*Cd4*^cre^ mice were subcutaneously injected with 2 × 10^5^ B16F10 cells and harvested after 13 days. **(J)** Representative flow cytometry plots and cumulative data show the frequency of Tcf1^+^ Tox^+^ cells gated on tumor-infiltrating CD8^+^ CD44^+^ T cells (right). *n* = 6 per group. **(K)** Schematic diagram of the OT-I–transferred tumor model: CD45.1 mice were subcutaneously injected with 2 × 10^5^ EG7-OVA cells, followed by 2 × 10^6^ WT or *Mettl8*^−/−^ OT-I cells transfer at 9 dpi. Mice were harvested at 21 dpi. Representative flow cytometry plots and cumulative data show the frequency of Tox^+^ cells gated on Tcf1^+^ OT-I cells. *n* = 6 per group. **(L)** The MFI of Tox gated on Tcf1^+^ OT-I cells of the mice in K. *n* = 6 per group. Data are representative of two independent experiments. P value was calculated by two-tailed Student’s *t* test; *P < 0.05; **P < 0.01; ****P < 0.0001. Source data are available for this figure: SourceData F5.

To investigate the molecular mechanism of Mettl8 regulating T_PEX_ cell transition, we conducted m^3^C-seq and Mettl8-RNA immunoprecipitation sequencing (RIP-seq) using sorted tumor-infiltrating CD8^+^ T cells. By co-analysis of Mettl8-binding genes in RIP-seq and downregulated genes in m^3^C and RNA-seq, we found eight genes regulated by Mettl8, including the T_PEX_ marker gene *Tcf7* (Fig. 5 A). Further analysis revealed that the m^3^C modification site is the same as the binding site of *Tcf7* mRNA (Fig. 5 B). Furthermore, *Mettl8* deficiency in CD8^+^ T cells accelerated the degradation of *Tcf7* mRNA (Fig. 5 C), suggesting that Mettl8 plays a crucial role in maintaining the stability of *Tcf7* mRNA.

To further investigate whether *Mettl8* deficiency causes alterations in Tcf1-binding sites, we performed Mettl8 and Tcf1 CUT&Tag in CD8^+^ T cells. The results revealed that the absence of Mettl8 significantly reduced the number of Tcf1-binding regions. Meanwhile, we found that 36% of the Tcf1-binding regions also showed Mettl8 binding (Fig. 5 D). These data suggested that Mettl8 not only maintained the stability of *Tcf7* mRNA but also interacted with *Tcf7* protein to regulate the gene program of stem-like T_PEX_ cells. Given that Tcf1 regulates genomic organization to maintain CD8^+^ T cell identity, we next examined whether *Mettl8* deficiency disrupts Tcf1-mediated 3D genome organization, leading to abnormal gene expression. Surprisingly, *Mettl8* deficiency resulted in the loss of a chromatin loop containing the *Tox* gene (Fig. 5 E), causing a reduction in *Tox* expression level. This finding is consistent with the observation that Tcf1/Lef1 KO diminishes the loop containing the *Tox* gene (Shan et al., 2021) (Fig. S4 F). Therefore, these results suggest that *Mettl8* deficiency disrupts the chromatin loop regulating *Tox* gene expression by affecting Tcf1-mediated interactions.

To further confirm the interaction between Mettl8 and Tcf1, co-immunoprecipitation (co-IP) was performed using CD3^+^ T cells from Mettl8-tdTomato-Flag mice, demonstrating the co-IP of Tcf1 with anti-Flag magnetic beads (Fig. 5 F). Additionally, Flag-tagged Mettl8 co-immunoprecipitated with Tcf1 when co-transferred into HEK293T cells (Fig. 5 G). Indeed, these data demonstrate that Mettl8 interacts with Tcf1 to maintain *Tox* gene expression.

The transcription factor Tox is a critical regulator of CD8^+^ T cell exhaustion (Khan et al., 2019; Scott et al., 2019; Yao et al., 2019), and Tcf1^+^ Tox^+^ stem-like T_PEX_ cells are found in the TME (Huang et al., 2022). To explore this further, we reanalyzed scRNA-seq data (Huang et al., 2022) and found that *Mettl8*-expressed cells were primarily *Tcf7* expressing and were enriched in Tcf1^+^ Tox^+^ stem-like T cells (Fig. 5 H). Other stem-like genes, such as *Slamf6*, *Cxcr5*, and *Bcl6*, were also expressed in Tcf1^+^ Tox^+^ stem-like T cells (Fig. 5 H). In contrast, effector and exhausted genes, including *Cx3cr1* and *Havcr2*, showed inverse expression patterns relative to Mettl8 (Fig. S4 G). *Pdcd1*, an exhaustion-related gene, was mainly expressed in Tox^+^ cells, including stem-like Tcf1^+^ Tox^+^ T cells (Fig. S4 G). Furthermore, *Mettl8* deficiency led to a reduction in Tcf1^+^ Tox^+^ stem-like T cells in both polyclonal (Fig. 5, I and J) and monoclonal (Fig. 5 K) CD8^+^ T cell populations. These stem-like T cells expressed lower levels of CX3CR1 and Tim3 and comparable levels of PD-1 compared with Tcf1^−^ Tox^+^ effector T/T_EX_ cells (Fig. S4 H). Additionally, Tox expression was significantly decreased in *Mettl8*-deficient cells (Fig. 5 L). Collectively, these data suggest that Mettl8 positively regulates Tox expression via Tcf1 in Tcf1^+^ Tox^+^ stem-like T cells.

### Ginkgolic acid (GA) suppresses Mettl8 to drive CD8^+^ T cell differentiation toward an effector state

**Figure 6.**
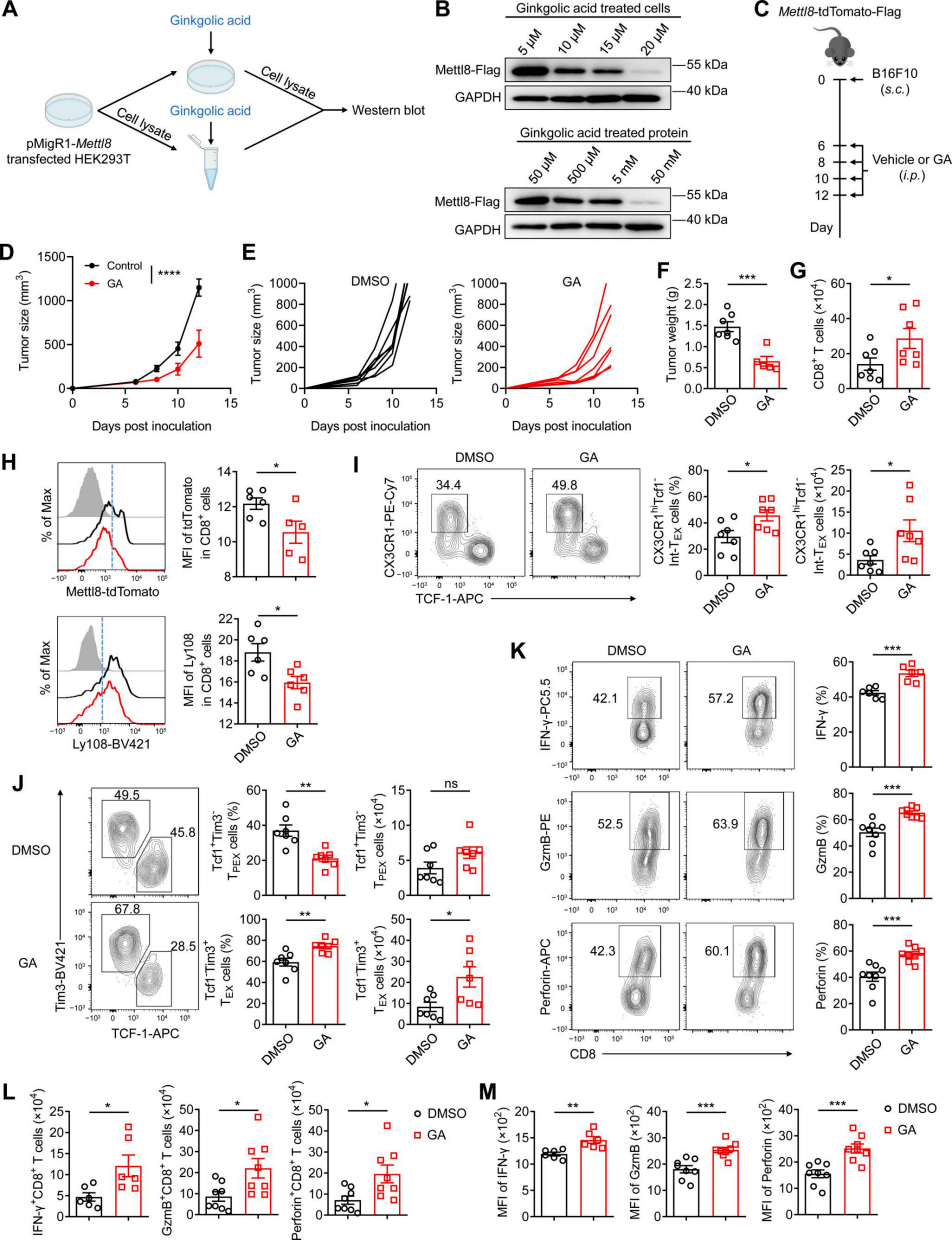
**GA enhances the antitumor response of CD8** ^**+**^ **T cells. (A)** Schematic diagram of detecting Mettl8 expression *in vitro*: HEK293T cells were transfected with pMigR1-Mettl8-Flag plasmid, and Flag-tagged Mettl8 was detected by western blotting. **(B)** The detection included GA-treated HEK293T cells after transfection (top) or treated protein extracted from transfected HEK293T cells (bottom). **(C)** Schematic diagram of GA treatment to B16F10 tumor–bearing *Mettl8*-tdTomato-Flag mice: Mice were subcutaneously injected with 2 × 10^5^ B16F10 cells, followed by GA treatment every 2 days from day 6 to day 12. Mice were harvested at day 13. **(D)** Tumor growth of the mice in C. *n* = 7 per group. **(E)** Tumor growth of the mice in C displayed in each replicate. **(F and G)** Tumor weight (F) and the absolute number of tumor-infiltrating CD44^+^ CD8^+^ T cells (G). *n* = 7 per group. **(H)** Representative flow cytometry plots (left) and cumulative data (right) show the MFI of tdTomato and Ly108 in tumor-infiltrating CD8^+^ T cells. *n* = 5–6 per group. **(I and J)** Representative flow cytometry plots and cumulative data show the frequency and absolute number of CX3CR1^+^ Tcf1^−^ Int-T_EX_ (I), Tcf1^+^ Tim3^−^ T_PEX_, and Tim3^+^ Tcf1^−^ T_EX_ cells (J) gated on tumor-infiltrating CD44^+^ CD8^+^ T cells. *n* = 7 per group. **(K)** Representative flow cytometry plots (left) and cumulative data (right) show the frequency of GzmB, IFN-γ, and perforin gated on tumor-infiltrating CD44^+^ CD8^+^ T cells. *n* = 6–8 per group. **(L and M)** Cumulative data show the absolute number (L) and MFI (M) of IFN-γ, GzmB, and perforin gated on tumor-infiltrating CD44^+^ CD8^+^ T cells. *n* = 6–8 per group. Data are representative of three independent experiments. P value was calculated by two-way ANOVA (D) and two-tailed Student’s *t* test (F–M); *P < 0.05; **P < 0.01; ***P < 0.001; ****P < 0.0001. Source data are available for this figure: SourceData F6.

A recent study has reported that the nuclear localization of Mettl8 is maintained by SUMOylation, a process that can be inhibited by GA, a known SUMO ligase inhibitor (Zhang et al., 2020). To validate the effect of GA on Mettl8 protein stability, we transfected HEK293T cells with a pMigR1-Mettl8-Flag plasmid. Treatment with increasing concentrations of GA resulted in a dose-dependent decrease in Mettl8 protein levels (Fig. 6, A and B), suggesting that GA disrupts Mettl8 protein stability.

Considering that GA did not affect tumor cell growth directly (Fig. S4 I), we administered GA to *Mettl8*^tdTomato^ mice bearing the subcutaneous B16F10 tumors (Fig. 6 C). GA treatment significantly suppressed tumor growth (Fig. 6, D–F). In tumor-infiltrating CD8^+^ T cells, GA reduced both Mettl8-tdTomato and the stem-like marker Ly108 (Fig. 6 H), indicating that Mettl8 inhibition diminishes stem-like features. Furthermore, GA increased the activated CD8^+^ T cells (Fig. 6 G) and induced a subset redistribution that mirrored the phenotype of Mettl8 deficiency: a marked reduced proportion of T_PEX_ subsets and a concomitant increase in Int-T_EX_ and T_EX_ subsets (Fig. 6, I and J). Consistently, GA enhanced cytokine production in CD8^+^ T cells, including IFN-γ, GzmB, and perforin, similar to observations in Mettl8-deficient OT-I cells (Fig. 6, K–M). These results demonstrate that GA suppresses tumor growth by promoting the differentiation of CD8^+^ T cells toward effector-like states and enhancing their cytokine secretion.

**Figure 7.**
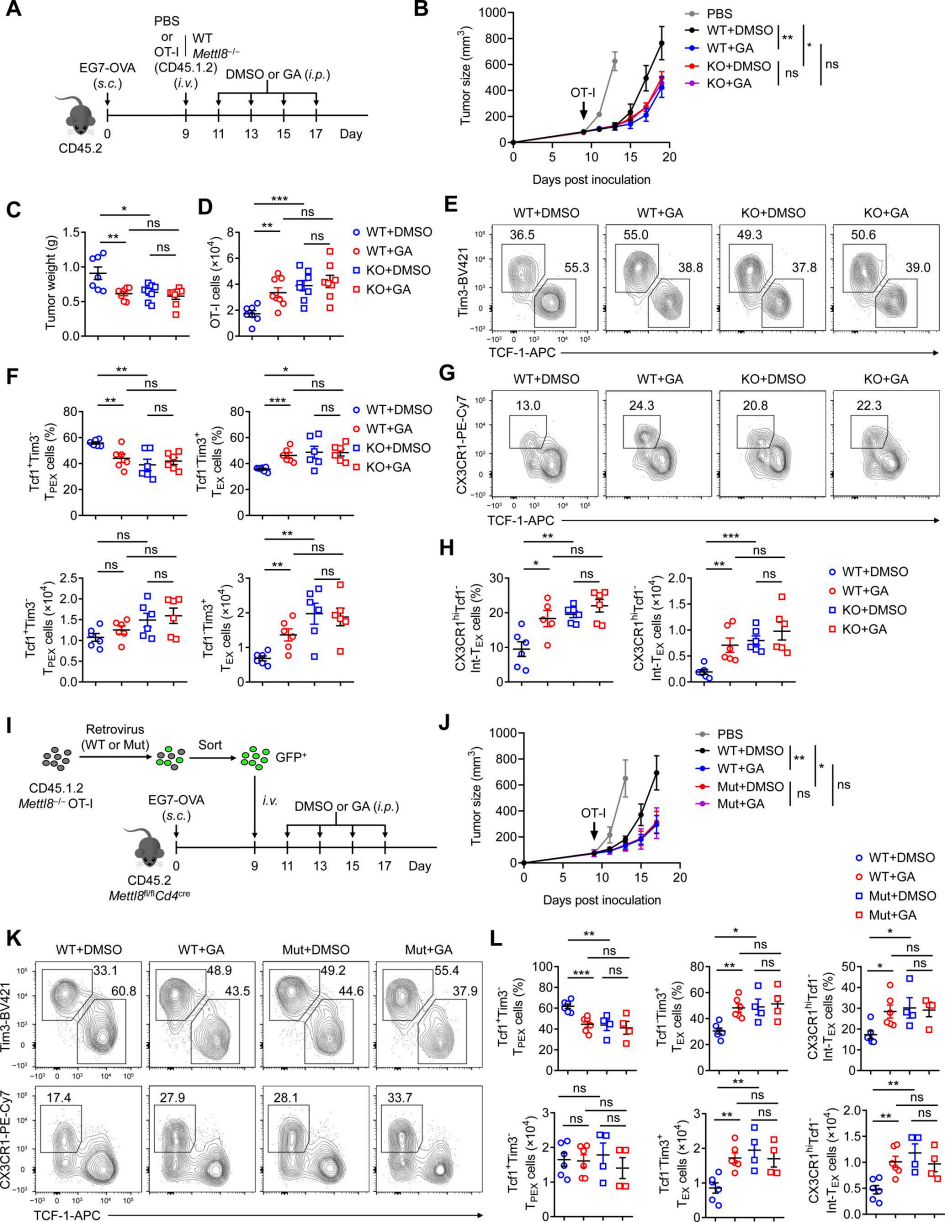
**GA potentiates CD8** ^**+**^ **T cell antitumor immunity via Mettl8 inhibition. (A)** Schematic diagram of GA-treated OT-I–transferred model: CD45.2 mice were subcutaneously injected with 2 × 10^5^ EG7-OVA cells, followed by 5 × 10^5^ CD45.1.2 WT or *Mettl8*^−/−^ OT-I cells transfer at 9 dpi. GA was administered i.p. every 2 days from 11 to 17 dpi. Mice were harvested at 19 dpi. **(B–D)** Tumor growth (B), tumor weight (C), and absolute number of tumor infiltrating OT-I cells (D) of the mice in A. *n* = 7–8 per group. **(E and F)** Representative flow cytometry plots (E) and cumulative data (F) show the frequency and absolute number of Tcf1^+^ Tim3^−^ T_PEX_ and Tim3^+^ Tcf1^−^ T_EX_ cells gated on tumor-infiltrating OT-I cells. *n* = 6 per group. **(G and H)** Representative flow cytometry plots (G) and cumulative data (H) show the frequency and absolute number of CX3CR1^+^ Tcf1^−^ Int-T_EX_ cells gated on tumor-infiltrating OT-I cells. *n* = 6 per group. **(I)** Schematic diagram of Mettl8-mutated mouse model: CD45.2 *Mettl8*^fl/fl^*Cd4*^cre^ mice were subcutaneously injected with 2 × 10^5^ EG7-OVA cells. Mettl8-WT or Mettl8-mutation (Mut) retrovirus were transduced to CD45.1.2 *Mettl8*^−/−^ OT-I cells. 5 × 10^5^ GFP^+^ cells were sorted 48 h after transduction and adoptively transferred into the tumor-bearing mice at 9 dpi. GA was administered i.p. every 2 days from 11 to 17 dpi. Mice were harvested at 18 dpi. **(J)** Tumor growth of the mice in I. *n* = 4–6 per group. **(K and L)** Representative flow cytometry plots (K) and cumulative data (L) show the frequency and absolute number of Tcf1^+^ Tim3^−^ T_PEX_, Tim3^+^ Tcf1^−^ T_EX_, and CX3CR1^+^ Tcf1^−^ Int-T_EX_ cells gated on tumor-infiltrating OT-I cells. *n* = 4–6 per group. Data are representative of two independent experiments. P value was calculated by two-way ANOVA (B and J) and two-tailed Student’s *t* test (C, D, F, H, and L); *P < 0.05; **P < 0.01; ***P < 0.001.

**Figure S5.**
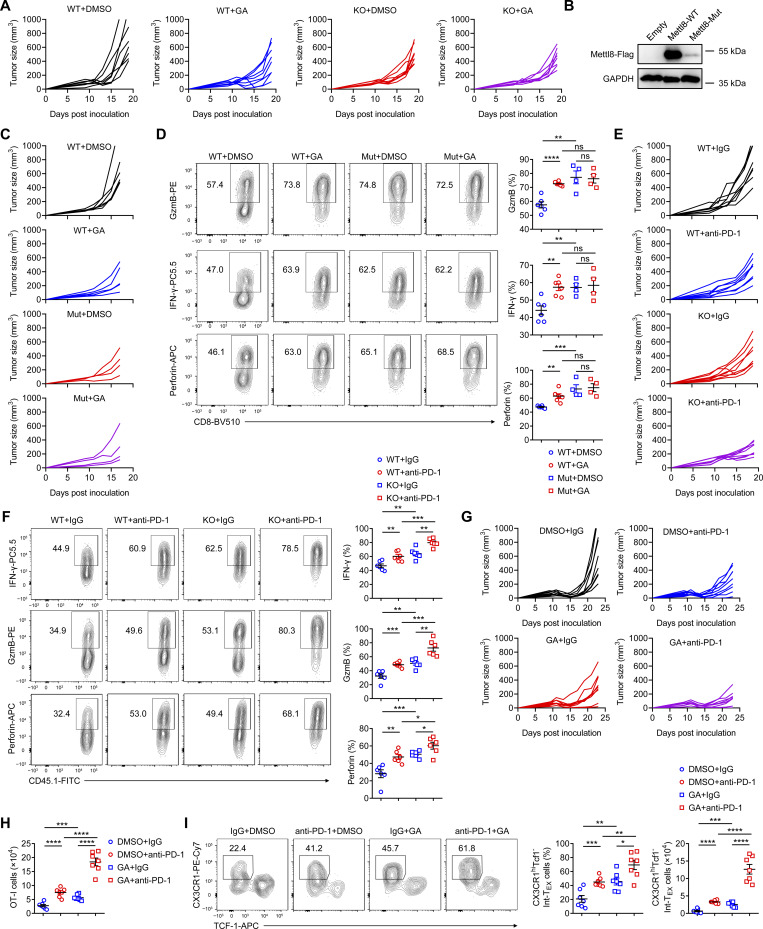
**Mettl8 inhibition promotes CD8** ^**+**^ **T cell antitumor immunity and synergistically enhances PD-1 blockade. (A)** Tumor growth of the mice in GA-treated adoptive-transferred model displayed in each replicate. *n* = 8 per group. **(B)** Western blot analysis of Mettl8-Flag and GAPDH in lysates from HEK293T cells transfected with pMIGR1-Empty, pMIGR1-Mettl8-WT, and pMIGR1-Mettl8-Mut plasmid. **(C)** Tumor growth curves for individual mice in the Mettl8-mutated mouse model. *n* = 4–6 per group. **(D)** Representative flow cytometry plots and cumulative data show the frequency of GzmB, IFN-γ, and perforin gated on tumor-infiltrating OT-I cells from the mice in C. *n* = 4–6 per group. **(E)** Tumor growth of the mice from the combined *Mettl8* KO and anti–PD-1 treatment model displayed in each replicate. *n* = 8 per group. **(F)** Representative flow cytometry plots and cumulative data show the frequency of IFN-γ, GzmB, and perforin gated on tumor-infiltrating OT-I cells from the mice in E. *n* = 6 per group. **(G)** Tumor growth of the mice from GA and anti–PD-1 combined treatment model displayed in each replicate. *n* = 9 per group. **(H)** Cumulative data show the absolute number of tumor-infiltrating OT-I cells from the mice in G. **(I)** Representative flow cytometry plots cumulative data show the frequency and absolute number of CX3CR1^+^ Tcf1^−^ Int-T_EX_ cells gated on tumor-infiltrating OT-I cells from the mice in G. *n* = 7 mice per group. P value was calculated by two-tailed Student’s *t* test; *P < 0.05; **P < 0.01; ***P < 0.001; ****P < 0.0001. Source data are available for this figure: SourceData FS5.

To further validate the tumor inhibition of GA is dependent on Mettl8 inhibition, we treated WT or *Mettl8*^−/−^ OT-I cell-transferred EG7-OVA subcutaneous tumor-bearing mice with GA or its DMSO vehicle (Fig. 7 A). GA treatment significantly suppressed tumor growth in mice receiving WT OT-I cells but had no effect in those receiving *Mettl8*^−/−^ OT-I cells (Fig. 7, B and C; and Fig. S5 A). Consistent with our previous findings, GA enhanced the accumulation of WT OT-I cells and promoted their differentiation toward Int-T_EX_ and T_EX_ subsets. In contrast, GA treatment did not alter the abundance or subset distribution of *Mettl8*^−/−^ OT-I cells (Fig. 7, D–H). These results suggest that GA-mediated tumor control and CD8^+^ T cell subset redistribution are strictly dependent on the presence of Mettl8.

Given that the SUMOylation site of Mettl8 is a lysine conserved across species (Zhang et al., 2020), we substituted this lysine with arginine in the pMigR1-Mettl8-Flag plasmid. This point mutation dramatically reduced the protein level of Mettl8 (Fig. S5 B), suggesting that the loss of SUMOylation leads to instability of the Mettl8 protein.

We next packaged lentiviruses using either the Mettl8-mutant (Mut) or Mettl8-WT plasmid and transduced *Mettl8*^−/−^ OT-I cells. The successfully transduced GFP^+^ cells were sorted and transferred into mice bearing EG7-OVA subcutaneous tumors, followed by GA treatment (Fig. 7 I). The Mettl8 mutation significantly enhanced tumor control, and GA treatment failed to provide additional inhibition in the mutant group (Fig. 7 J and Fig. S5 C). Analysis of OT-I cell subsets revealed that the mutation promoted differentiation toward an effector/exhausted state (Fig. 7, K and L) and upregulated the proportion of effector molecules (Fig. S5 D). Collectively, these results demonstrate that GA drives CD8^+^ T cells toward an effector-like state by suppressing Mettl8 via inhibition of its SUMOylation.

### Mettl8 inhibition enhances the efficacy of anti–PD-1 therapy

**Figure 8.**
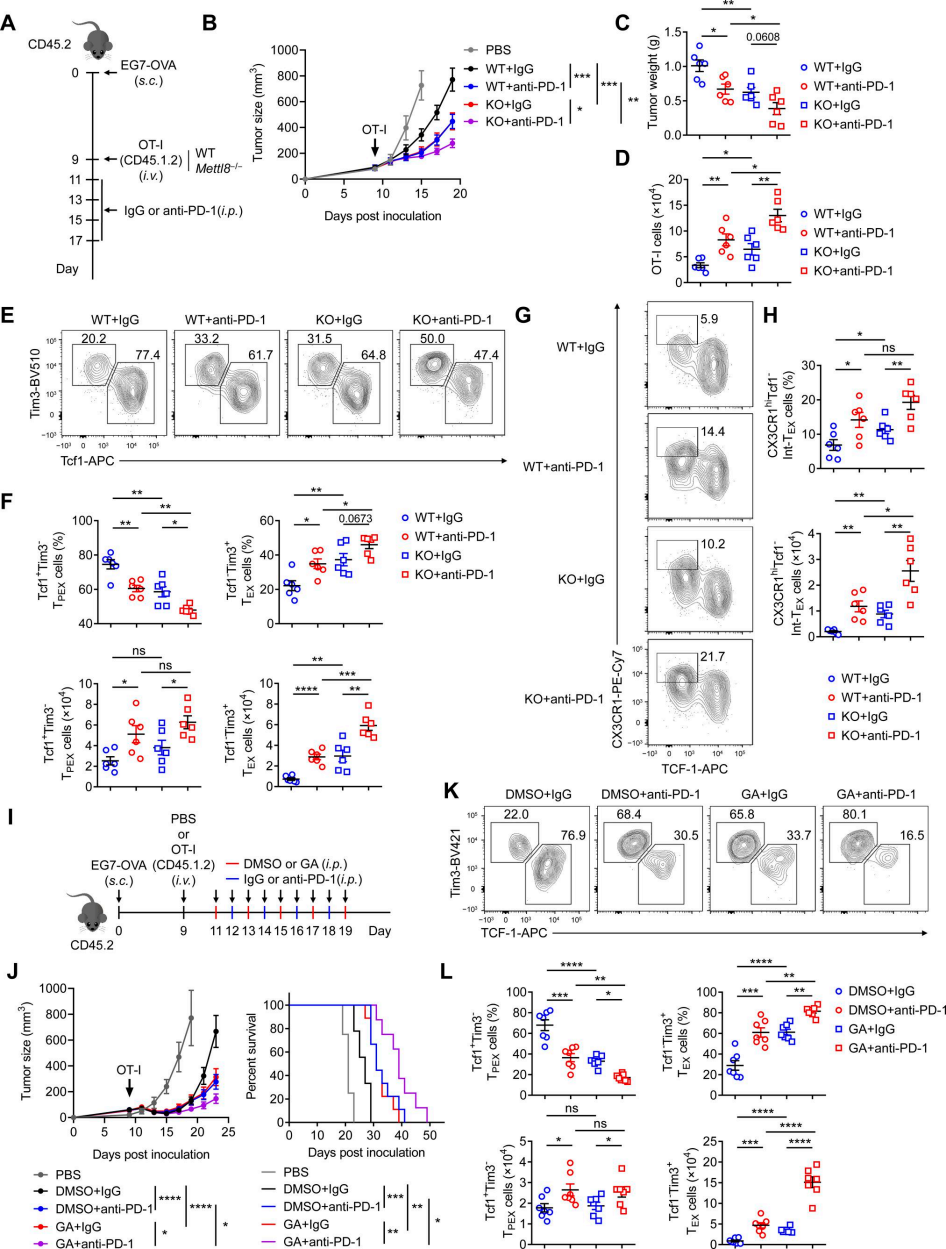
**Mettl8 inhibition synergizes with anti–PD-1 treatment. (A)** Schematic diagram of the combined *Mettl8* KO and anti–PD-1 treatment model: CD45.2 mice were subcutaneously injected with 2 × 10^5^ EG7-OVA cells, followed by 5 × 10^5^ CD45.1.2 WT or *Mettl8*^−/−^ OT-I cells transfer at 9 dpi. Anti–PD-1 antibody was administered i.p. every 2 days from 11 to 17 dpi. Mice were harvested at 19 dpi. **(B–D)** Tumor growth (B), tumor weight (C), and absolute number of tumor infiltrating OT-I cells (D) of the mice in A. *n* = 6 per group. **(E and F)** Representative flow cytometry plots (E) and cumulative data (F) show the frequency and absolute number of Tcf1^+^ Tim3^−^ T_PEX_ and Tim3^+^ Tcf1^−^ T_EX_ cells gated on tumor-infiltrating OT-I cells. *n* = 6 per group. **(G and H)** Representative flow cytometry plots (G) and cumulative data (H) show the frequency and absolute number of CX3CR1^+^ Tcf1^−^ Int-T_EX_ cells gated on tumor-infiltrating OT-I cells. *n* = 6 per group. **(I)** Schematic diagram of the combined GA and anti–PD-1 treatment model: CD45.2 mice were subcutaneously injected with 2 × 10^5^ EG7-OVA cells, followed by 2 × 10^6^ CD45.1.2 OT-I cell transfer at 9 dpi. GA and anti–PD-1 antibody were administered i.p. in an alternating schedule from 11 to 19 dpi. Mice were harvested at 23 dpi. **(J)** Tumor growth (left) and survival curve (right) in each group of the mice in I. *n* = 8–9 per group. **(K and L)** Representative flow cytometry plots (K) and cumulative data (L) show the frequency and absolute number of Tcf1^+^ Tim3^−^ T_PEX_ and Tim3^+^ Tcf1^−^ T_EX_ cells gated on tumor-infiltrating OT-I cells. *n* = 6–7 per group. Data are representative of two independent experiments. P value was calculated by two-way ANOVA (B and J), two-tailed Student’s *t* test (C, D, F, H, and L), and Log-rank test (J); *P < 0.05; **P < 0.01; ***P < 0.001; ****P < 0.0001.

To evaluate the effect of *Mettl8* deficiency on anti–PD-1 therapy, we treated WT or *Mettl8*^−/−^ OT-I cell-transferred EG7-OVA tumor-bearing mice with anti–PD-1 antibody (Fig. 8 A). While either anti–PD-1 treatment or Mettl8 deficiency alone delayed tumor growth, their combination resulted in significantly superior tumor suppression (Fig. 8, B and C; and Fig. S5 E). Similarly, the number of tumor-infiltrating OT-I cells increased with either treatment and was highest in the combination group (Fig. 8 D). Subset analysis revealed that anti–PD-1 treatment reduced the frequency of T_PEX_ cells with slightly increase in their absolute number, while concurrently expanding the Int-T_EX_ and T_EX_ subsets. This skew toward a more effector-like state was further potentiated in *Mettl8*^−/−^ OT-I cells upon anti–PD-1 treatment (Fig. 8, E–H). Consistently, the production of effector molecules was highest in the combination group (Fig. S5 F). These results demonstrate that Mettl8 deficiency synergizes with anti–PD-1 therapy to enhance antitumor immunity.

Given the therapeutic effect of Mettl8 inhibitor GA, we next explored its potential synergy with anti–PD-1 therapy (Fig. 8 I). The combination of GA and anti–PD-1 led to significantly improved tumor control compared with either monotherapy (Fig. 8 J and Fig. S5 G). This enhanced antitumor effect was associated with a greater expansion of OT-I cells (Fig. S5 H) and a pronounced shift in their differentiation toward Int-T_EX_ and T_EX_ subsets (Fig. 8, K and L; and Fig. S5 I). These findings demonstrate that targeting Mettl8 with GA synergizes with PD-1 blockade to augment T cell antitumor immunity.

## Discussion

In this study, we identified *Mettl8*, an m^3^C methyltransferase, as a critical regulator of CD8^+^ T cell–mediated antitumor immunity. Mettl8 expression was significantly higher in T_PEX_ cells compared with T_EX_ cells and decreased in response to anti–PD-1 treatment. Conditional KO of Mettl8 in T cells led to an increase in Int-T_EX_ and T_EX_ cells and enhanced secretion of effector molecules. Mechanistically, our findings supported that Mettl8 promotes m^3^C modification by binding to *Tcf7* mRNA, thereby maintaining its stability. Additionally, Mettl8 interacts with Tcf1 protein to regulate downstream genomic 3D structures. Building on these insights, we utilized the Mettl8 inhibitor GA to treat tumors in mouse models. This treatment significantly enhanced CD8^+^ T cell functionality and effectively suppressed tumor growth. These results highlight Mettl8 as a potential therapeutic target for enhancing antitumor immunity.

Reversing T cell exhaustion is a critical challenge in cancer immunotherapy. During the exhaustion process, stem-like T_PEX_ cells transit into effector-like Int-T_EX_ cells and eventually terminal T_EX_ cells, resulting in functional decline (Chu et al., 2025; McManus et al., 2025). Current research suggests that T_EX_ cells are continuously replenished through the differentiation of stem-like T_PEX_ cells via Int-T_EX_ intermediates, while T_PEX_ cells maintain their function through self-renewal (Miller et al., 2019; Siddiqui et al., 2019). Tcf1 is a key transcription factor essential for the self-renewal of stem-like progenitor exhausted CD8^+^ T cells (Chen et al., 2019; Wu et al., 2016). Recent studies have identified several regulators of Tcf1, including the m^6^A methyltransferase METTL3 (Yao et al., 2021b), the chromatin regulator Mll1 (Bélanger et al., 2023), intrinsic HDAC domain (Li et al., 2020; Xing et al., 2016), and disordered region (Goldman et al., 2023). Additionally, novel research has demonstrated that Tcf1 is essential for 3D genome reconfiguration (Wang et al., 2022), with Tcf1 and CTCF collaboratively promoting chromatin interactions to organize genomic architecture (Shan et al., 2022).

Our study revealed that Mettl8 deficiency led to a reduction in Tcf1 expression, which adversely affected the 3D structure of the Tox genomic region, thereby reducing Tox expression. This shift promoted the differentiation of T_PEX_ cells into Int-T_EX_ cells with enhanced effector functions while reducing their progression toward a dysfunctional state. These findings suggest that inhibiting Mettl8 can preserve the effector function of T_EX_ cells, offering a promising new avenue for improving cancer immunotherapy.

A recent study reported that Mettl8 formed a large SUMOylated nuclear RNA-binding protein complex, and GA, a known SUMO ligase inhibitor, completely abolished Mettl8 SUMOylation (Zhang et al., 2020). In our study, treatment with GA demonstrated significant tumor growth inhibition. Notably, tumor-infiltrating CD8^+^ T cells exhibited increased expression of effector molecules such as IFN-γ and perforin, accompanied by downregulation of Mettl8 expression. Furthermore, *in vitro* treatment of Mettl8-overexpressing HEK293T cells or their protein extracts with GA showed dose-dependent inhibition of Mettl8. However, the SUMOylation status of Mettl8 was not assessed, leaving it unclear whether the inhibition of Mettl8 by GA was due to the abolition of its SUMOylation. Thus, further investigation is needed to determine whether GA inhibits Mettl8 SUMOylation in CD8^+^ T cells, which could provide deeper insights into its mechanism of action and potential therapeutic applications.

## Materials and methods

### Mice

*Mettl8* ^fl/fl^ mice were generated by Shanghai Biomodel Organism Center, China. *Mettl8*-tdTomato-Flag mice were generated by Cyagen Biosciences, China. The C57BL/6J (B6, CD45.2), B6.SJL (CD45.1), *Cd4*^cre^, *Gzmb*^cre^, P14, and OT-I mice were from the Jackson Laboratory. For this study, mice analyzed were 6–10 wk of age, and both genders were used without randomization or blinding. All mice were housed in a specific pathogen–free facility in accordance with the guidelines for experimental animals at the University of Science and Technology of China. All experimental procedures involving mice were approved by the Ethics Committee of the University of Science and Technology of China and conducted following the National Guidelines for Animal Usage in Research (China).

### Cell lines

The MC38, B16F10, EG7-OVA, and HEK293T were purchased from the cell bank of the Chinese Academy of Sciences (Shanghai, China). All cell lines were mycoplasma free. MC38 and HEK293T were cultured in DMEM medium (Hyclone). B16F10 and EG7-OVA were cultured in RPMI-1640 medium (Hyclone). All the media were supplemented with 10% FBS (BI) and 1% penicillin-streptomycin solution (Gibco).

### Tumor models

For adoptive transfer tumor model, EG7-OVA cells (2 × 10^5^) were injected subcutaneously into recipients. Spleen cells of OT-I mice were isolated and cultured at 1.5 × 10^6^ per milliliter in RPMI-1640 medium with 10% FBS, 1% penicillin-streptomycin, and 2-mercaptoethanol and supplemented with 10 nmol/L OVA257–264 peptide and 10 ng/mL human recombinant IL-2 for 3 days. Then the cells were cultured in fresh medium containing 100 U/mL IL-2 for 2 more days before transfer. Tumor-bearing mice with similar tumor size were randomly divided into specific groups and, respectively, received PBS, WT OT-I cells, or Mettl8^−/−^ OT-I cells (5 × 10^5^ or 2 × 10^6^) i.v. injection. Tumor size was calculated as length × width × width/2 every 2 days. When the tumor size was larger than 1,000 mm^3^, the mice were euthanized for ethical consideration.

For melanoma subcutaneous tumor model, mice were injected subcutaneously with 2 × 10^5^ B16F10 cells in 100 μL of PBS. Mice were sacrificed at 14 days after inoculation. For colorectal cancer liver metastasis model, mice were injected intrasplenically with 2 × 10^5^ MC38 cells in 50 μL of PBS, followed by splenectomy 3 min after injections. Mice were sacrificed 21 days after the initial injection. For melanoma lung metastasis model, mice were injected i.v. with 2 × 10^5^ B16F10 cells in 500 μL of PBS. Mice were monitored for survival. Tumor size was calculated as length × width × width/2 every 2 days. When the tumor size was larger than 1,000 mm^3^, the mice were euthanized for ethical consideration.

### Retroviral constructs and transduction

*Mettl8* cDNA was cloned into a GFP-expressing retroviral vector pMigR1 (cat. no. #27490; Addgene). Retrovirus was packaged in HEK293T cells as previously described (Li et al., 2018). Splenocytes from *Mettl8*^−/−^ or littermate control OT-I TCR-transgenic mice were cultured in RPMI-1640 medium supplemented with 10% FBS, 1% penicillin-streptomycin, β-mercaptoethanol, 5 μg/mL anti-CD3, and 2.5 μg/mL anti-CD28. Cells were plated in 24-well plates at a density of 2.5 × 10^6^ cells/mL and stimulated for 20–24 h. Subsequently, cells were transduced with retrovirus by spinofection (2,500 rpm, 32°C for 90 min), incubated for an additional 3 h, and then transferred from one well of a 24-well plate to a 10-cm culture dish. After 48 h of culture, GFP-positive cells were isolated by flow cytometry sorting.

### Adoptive transfer and LCMV infection

Naïve P14 CD8^+^ T cells were isolated from the LNs from *Mettl8*^−/−^ and littermate control P14 TCR-transgenic mice. 1 × 10^4^ P14 cells were transferred followed by i.v. infection with 2 × 10^6^ PFU of LCMV-clone13 24 h after transfer. Mice were harvested 30 days after infection.

### GA treatment

GA was purchased from MedChemExpress and suspended in DMSO to 20 mg/mL for storage. For *in vivo* treatment, GA was diluted to 20 μg/mL in solvent containing 5% DMSO, 40% PEG300, 5% Tween-80, and 50% PBS. For B16F10 subcutaneous tumor model, GA was i.p. injected 200 μg per mouse every 2 days from day 6 to 12 after inoculation. For adoptively transferred tumor model, GA was i.p. injected 200 μg per mouse every 2 days from day 11 to 17 after inoculation. For *in vitro* treatment, 293T cells were transfected with pMigR1-Mettl8 plasmid described above. GA was added to the cell medium 12 h after transfection, followed by cell lysis 24 h later, or added to the cell lysate 36 h after transfection, followed by standing in 4°C for 24 h. For B16F10 cell treatment *in vitro*, 0, 5, or 10 μM GA was added to the cell medium. After 24 h, cells were collected for cell count and 7-AAD test by flow cytometry.

### Anti–PD-1 antibody treatment

Anti–PD-1 treatments involved i.p. administration of 200 μg per mouse of anti–PD-1 antibody (clone RMP1-14, cat. no. A2122; Selleckchem) or rat IgG2a isotype control (clone 2A3, cat. no. BE0089; Bio X Cell) in 200 μL PBS every 2 days from day 11 to 17 or day 12 to 18 after inoculation.

### *In vivo* suppression assay

For the *in vivo* suppression assay, spleen naïve CD4^+^ T_conv_ cells (TCRβ^+^ CD4^+^ CD45RB^+^ CD25^–^) were sorted from CD45.1^+^ mice; spleen T_reg_ cells (TCRβ^+^ CD4^+^ YFP^+^) were sorted from WT or *Mettl8*^fl/fl^*Cd4*^cre^ mice. A total of 4 × 10^5^ naive CD4^+^ Tconv cells were transferred i.v. alone or together with 2 × 10^5^ WT or *Mettl8*^fl/fl^*Cd4*^cre^ T_reg_ cells into *Rag1*^−/−^ recipients. Recipient mice were monitored and weighed every 2–3 days after transfer for signs of disease, such as weight loss.

### Preparation of single-cell suspensions, antibody staining, and flow cytometry

To obtain tumor tissue-infiltrating cells, the mice were euthanized, and the tumors were carefully removed, cut into small pieces using surgical scissors mechanically. The tumors were digested in DMEM medium supplemented with 1 mg/mL collagenase type IV and 10 U/mL DNase I for 40 min at a 37°C shaking incubator (200 rpm). Leukocytes were obtained by suspension in 40% Percoll and density gradient centrifugation. The cell suspension was then filtered using a cell strainer (70 μm). Isolated cells were used for various assays. To obtain liver mononuclear cells (MNCs), the tissue was passed through a 200-gauge mesh. The obtained cells were resuspended in 40% Percoll (GE Healthcare) and overlaid carefully onto 70% Percoll. After centrifugation, liver MNCs were collected from the interphase. The spleen, LNs, and thymus were crushed through a 70-μm cell strainer, and RBCs were lysed with RBC lysis buffer. To stain intracellular cytokines, cells were incubated for 4 h with 50 ng/mL PMA (Sigma-Aldrich) and 1 μg/mL ionomycin (Sigma-Aldrich) in the presence of 2.5 μg/mL monensin at 37°C in RPMI 1640 medium containing 10% FBS.

Single-cell suspensions were stained with fluorochrome-conjugated antibodies as described (Song et al., 2022). The fluorochrome-conjugated antibodies were as follows: anti-CD44 (IM7), anti-CD62L (MEL-14), anti-CD69 (H1.2F3), anti-CD24 (M1/69a), anti-CD25 (PC61.5), anti-TCRβ (H57-597), anti-Ki67 (SolA15), anti–PD-1 (J43), anti-Tim3 (RMT3-23), and rat IgG2a κ isotype control (eBR2a, for intracellular staining of Bcl6) were from eBioscience; anti-Tcf1 (C63D9) and isotype control (cat. no. 4410S for intracellular staining of Tcf1) were from Cell Signaling Technology; anti-CD45.2 (104), anti-CD45.1 (A20), anti-CXCR5 (J252D4), anti-CX3CR1 (SA011F11), anti-Granzyme B (QA16A02), anti-IFN-γ (XMG1.2), and anti-Perforin (S16009A) were from BioLegend; anti-CD4 (RM4-5), anti-CD8 (53-6.7), anti-Ly108 (13G3), and anti-Bcl6 (K112-91) were from BD Biosciences. For detection of transcription factors, surface-stained cells were fixed and permeabilized with the Foxp3/Transcription Factor Staining Buffer Set (eBioscience), followed by incubation with corresponding fluorochrome-conjugated antibodies. Flow cytometric acquisition was performed immediately. Data were collected on the Beckman CytoFLEX and were analyzed with FlowJo software version 10 (TreeStar).

### Immunoblotting

The protein expression in cells was determined by western blotting analysis as described (Song et al., 2021). Primary antibodies against the following proteins were used: anti-Flag (1:2,000, clone M2, Sigma-Aldrich), anti-Mettl8 (1:1,000, polyclonal, Novus), anti-β-actin (1:1,000, Cell Signaling Technology), and anti-GAPDH (1:100,000, cat. no. AC033; Erpan Tech). HRP-linked, anti-mouse (1:3,000, cat. no. 58802S; Cell Signaling Technology) or anti-rabbit (1:3,000, cat. no. 7074P2; Cell Signaling Technology) secondary antibodies were used. Signals were imaged using GE ImageQuant LAS 4000 or Tanon 5200.

### co-IP

A 3×Flag tag was fused to the N-terminus pMigR1-*Mettl8* plasmid on N-terminal of Mettl8 cDNA. The pMigR1-*Mettl8*-Flag together with pMigR1-*Tcf7* plasmids were transfected into 293T cells using Lipofectamine 2000 (Life Technologies), and 48 h later, cell lysates were extracted and incubated overnight with 10 μL of anti-FLAG magnetic beads (clone M2, Sigma-Aldrich). Magnetic beads were separated from the solution using a magnetic stand. After proper washing, the immunoprecipitated samples were analyzed by immunoblotting with anti-Tcf1 (clone C46C7, Cell Signaling Technology). The cell lysates were probed with anti-Tcf1 or anti-Flag to detect input proteins.

### Quantitative PCR with RT (RT-qPCR)

CD3^+^ T cells from the spleen of *Mettl8*^fl/fl^*Cd4*^cre^ mice and littermate controls were magnetic-activated cell sorted (MACS) using magnetic beads (Miltenyi Biotec) as per the manufacturer’s instructions. Cells were pelleted, and RNA was isolated with the Direct-zol RNA MiniPrep kit (cat. no. R2052; Zymo Research). RNA was quantified by NanoDrop spectrophotometers and then reverse transcribed into cDNA by using the HiScript III first strand cDNA synthesis kit (cat. no. R312-02; Vazyme) in A200 Gradient Thermal Cycler (LongGene). RT-qPCR was performed using SYBR qPCR Master Mix (cat. no. Q711-02; Vazyme) in qTOWER^3^ G Real-Time Thermal Cyclers (Analytik Jena). The expression of each gene was normalized to β-actin. RT-qPCR primers are as follows: *Mettl8*-F (5′–3′) 5′-GAA​GAA​GAA​GAC​GC-AGC​TAG​AA-3′; *Mettl8*-R (5′–3′) 5′-ATC​CCA​GTA​TTT​GTT​AGC​GTC-3′; *Actb*-F (5′–3′) 5′-CAT​TGC​TG-ACA​GGA​TGC​AGA​AGG-3′; *Actb*-R (5′–3′) 5′-TGC​TGG​AAG​GTG​GAC​AGT​GAG​G-3′.

### RNA decay assay

CD8^+^ T cells from the spleens of *Mettl8*^fl/fl^*Cd4*^cre^ mice and littermate controls were purified by MACS. 5 × 10^5^ purified CD8^+^ T cells in RPMI 1640 medium supplemented with 10% FBS, 100 U/mL of IL-2, 5 ng/mL CD3, and 2.5 ng/mL CD28 were seeded into 48-well plates. Meanwhile, actinomycin D (MedChemExpress) was added to a final concentration of 10 μM, and cells were harvested at t = 0, 0.5, 1, 1.5, 2, 2.5, and 3 h after actinomycin D treatment. Total RNAs were extracted and subjected to RT-qPCR analysis. Primers used are as follows: *Tcf7*-F (5′–3′) 5′-CCT​GCG​GAT​ATA​GAC​AGC​ACT​TC-3′; *Tcf7*-R (5′–3′) 5′-TGT​CCA​GGT​ACA​CCA​GAT​CCC​A-3′; *Gapdh*-F (5′–3′) 5′-CAT​CAC​TGC​CAC​CCA​GAA​GAC​TG-3′; *Gapdh*-R (5′–3′) 5′-ATG​CCA​GTG​AGC​TTC​CCG​TTC​AG-3′.

### scRNA-seq data analysis

The published scRNA-seq data are obtained from GEO: GSE260448, GSE216800, GSE124691, GSE116390, GSE121478, GSE86028, GSE122713, GSE137015, and GSE180094; and Zenodo at https://doi.org/10.5281/zenodo.12542577. The raw read count matrix, with genes as rows and cells as columns, was processed using the SCANPY pipeline for quality control and downstream analysis. For coding gene analysis, cells with >5% mitochondrial reads or <200 detected genes were excluded. Genes expressed in <3 cells were also filtered out. The count matrix was normalized using SCANPY’s normalize_total function, and the top 4,000 most highly variable genes were selected for principal component analysis (PCA). The first 30 principal components were then used to compute the UMAP for dimensionality reduction and visualization. For volcano plot, genes were considered significant at FDR <0.01.

### Bulk RNA-seq library construction

CD45.1 mice were subcutaneously injected with 2 × 10^5^ EG7-OVA cells, followed by 2 × 10^6^ WT or *Mettl8*^−/−^ OT-I cells transfer at 9 days post-inoculation (dpi). Mice were harvested at 21 dpi and CD45.2^+^ CD8^+^ OT-I cells from the tumors were sorted. RNA was extracted using the Direct-zol RNA MiniPrep kit (Zymo Research). A total amount of 1 μg RNA per sample was used as input material for the RNA sample preparations. Sequencing libraries were generated using NEBNext UltraTM RNA Library Prep Kit for Illumina (NEB) following the manufacturer’s recommendations, and index codes were added to attribute sequences to each sample. Briefly, mRNA was purified from total RNA using poly-T oligo-attached magnetic beads. Fragmentation was carried out using divalent cations under elevated temperature in NEBNext First Strand Synthesis Reaction Buffer (5×). First, strand cDNA was synthesized using random hexamer primer and M-MuLV Reverse Transcriptase (NEB). Second, strand cDNA synthesis was subsequently performed using DNA Polymerase I and RNase H. Remaining overhangs were converted into blunt ends via exonuclease/polymerase activities. After adenylation of 3′ ends of DNA fragments, NEBNext Adaptor with hairpin loop structure were ligated to prepare for hybridization. To select cDNA fragments of preferentially 250–300 bp in length, the library fragments were purified with AMPure XP system (Beckman Coulter). Then 3 μL USER Enzyme (NEB) was used with size-selected, adaptor-ligated cDNA at 37°C for 15 min, followed by 5 min at 95°C before PCR. Then PCR was performed with Phusion High-Fidelity DNA polymerase, Universal PCR primers, and Index (X) Primer. At last, PCR products were purified (AMPure XP system), and library quality was assessed on the Agilent Bioanalyzer 2100 system. The RNA-seq data are deposited at the Gene Expression Omnibus (GEO) under accession number GSE249068.

### Bulk RNA-seq data analysis

For bulk RNA-seq analysis, raw reads were aligned to the mm10 mouse reference genome (UCSC) using STAR (version 2.7.0a) with default parameters. Uniquely mapped reads were retained for gene-level quantification using the htseq-count function from the HTSeq package. Differential expression analysis was performed using DESeq2 in R. Genes with an adjusted P value (FDR) <0.05 and an absolute log_2_ fold change ≥2 were considered significantly differentially expressed. Data visualization included PCA, heatmaps, and volcano plots were generated using pheatmap, EnhancedVolcano, and ggplot2. DEGs’ function enrichment was using GSEA.

### m^3^C AlkAniline-seq library construction

Following the cell sorting strategy of RNA-seq, 1 × 10^7^ CD8^+^ T cells per sample were prepared. m^3^C AlkAniline-seq was performed by CloudSeq Biotech Inc. according to the published procedure with slight modifications (Marchand et al., 2018). Briefly, RNA samples were subjected to alkaline hydrolysis for fragmentation. RNA fragments were dephosphorylated with antarctic phosphatase (NEB) and then incubated in 1 M aniline for cleavage. RNA libraries were constructed with GenSeq Small RNA Library Prep Kit (GenSeq Inc.) by following the manufacturer’s instructions. Libraries were controlled for quality and quantified using the Bioanalyzer 2100 system (Agilent Technologies). High throughput sequencing was performed on an Illumina NovaSeq instrument.

### m^3^C AlkAniline-seq data analysis

Raw sequencing reads were first subjected to quality control using FastQC, and adapter trimming was performed with cutadapt (version 1.9.1). High-quality reads were then aligned to the mouse reference genome (mm10) using Bowtie2 with default parameters. Raw counts and coverage counts were calculated by bedtools (version 2.24) software and in-house scripts, and then ratio (defined as: count/coverage) and fc (defined as: treat-methyl-ratio/input-methyl-ratio) (Schwartz et al., 2014) were also calculated. Differentially m^3^C sites were calculated based on fc, with significant changes defined as FDR-adjusted P value <0.05 and fold change ≥2.

### RIP-seq library construction

*Mettl8*-tdTomato-Flag mice were inoculated with 2 × 10^5^ B16F10, and on day 13 after infection, CD8^+^ CD44^hi^ CD62L^lo^ T cells from the tumors were sorted. RIP-Seq service was provided by Cloud-Seq Biotech. Briefly, the RNA immunoprecipitation assay was carried out with GenSeq RIP kit (GenSeq Inc.) according to the manufacturer’s instructions. rRNAs were removed from the immunoprecipitated RNA and input RNA samples by using GenSeq rRNA Removal Kit (GenSeq, Inc.). RNA libraries were constructed by using rRNA-depleted RNAs with GenSeq Low Input RNA Library Prep Kit (GenSeq, Inc.) according to the manufacturer’s instructions. Libraries were controlled for quality and quantified using the Bioanalyzer 2100 system (Agilent Technologies, Inc.). Library sequencing was performed on an Illumina NovaSeq instrument with 150-bp paired-end reads.

### RIP-seq data analysis

Transcriptome high throughput sequencing of RIP-enriched RNA and subsequent bioinformatics analysis were all done by Cloud-Seq Biotech. Briefly, paired-end reads were harvested from Illumina NovaSeq 6000 sequencer and were quality controlled by Q30. After 3′ adapter-trimming and low-quality reads removing by cutadapt software (version 1.9.3), the high-quality reads were aligned to the reference genome with hisat2 software (version 2.0.4). Binding sites on RNAs (peaks) were identified by MACS2 software. Then, HTSeq software (version 0.9.1) was used to get the gene level raw count as the expression profiling, and edgeR (version 3.16.5) was used to perform normalization, and differentially expressed mRNAs were identified with significance defined as FDR-adjusted P < 0.05 and fold change ≥2.

### ATAC-seq library construction

Following the experimental condition and cell sorting strategy of RNA-seq, 1 × 10^5^ cells were prepared per sample, each in two biological replicates. Briefly, the sorted cell pellets were washed and treated in lysis buffer for 5 min on ice. The extracted nuclei were resuspended in fragmented buffer, including 1.5 μl transposome (Novoprotein N248), and incubated at 37°C for 30 min. The products were purified with tagment DNA extract beads (Novoprotein N245) and then amplified by PCR for 14 cycles with NovoNGS index kit for Illumina (N239). DNA fragments in the range of 200–800 bp were recovered from 2% E-Gel EX Agarose Gels (Invitrogen/Thermo Fisher Scientific). The libraries were sequenced on Illumina HiSeq sequencing systems in paired end 150 bp reads.

### ATAC-seq data analysis

For the ATAC-seq analysis, reads were aligned to the mm10 assembly of the mouse genome using Bowtie2 with the default parameter. Adapter sequences and low-quality bases were removed using cutadapt. Clean reads were aligned to the mouse reference genome (mm10) using Bowtie2. The ATAC peak calling was done by MACS2 with “-nomodel” and “-extsize 272” parameters. Signal tracks for genome browser visualization were generated using bam2wig, followed by conversion to BigWig format using UCSC tools. These tracks were loaded into IGV for inspection of peak quality and enrichment patterns. For differential binding analysis between experimental groups, read count matrices were constructed using DiffBind (R package), and significant differential ATAC peaks between groups were calculated with DESeq2 method (FDR < 0.05 and |log_2_ fold change| ≥1). Enrichment signals around transcription start sites (TSS ± 2 kb) were profiled using deepTools (computeMatrix, plotHeatmap).

### High-resolution chromosome-conformation capture library construction

Following the experimental condition and cell sorting strategy of RNA-seq, 1 × 10^6^ cells were prepared per sample, each in two biological replicates. High-resolution chromosome-conformation capture (Hi-C) was performed using the Hi-C 3.0 protocol as previously described (Lafontaine et al., 2021). In brief, the sorted cells were cross-linked with 1% formaldehyde for 10 min at 25°C. The cross-linked cells were lysed in 1 mL lysis buffer (10 mM Tris-HCl, pH 8.0, 10 mM NaCl, and 0.2% NP-40) supplemented with protease inhibitor cocktail (Millipore/Sigma-Aldrich) at 4°C for 15 min. The nuclei were collected with 900 μL 1×CutSmart buffer (NEB) and then treated with 50 μL 0.5% SDS at 62°C for 10 min, followed by adding 25 μL 10% Triton X-100 and 145 μl H_2_O to quench SDS. The resulting chromatin was then digested with NUB I (NEB) restriction enzymes at 37°C overnight. The reaction was stopped by standing on 65°C for 20 min. The DNA ends were blunted and labeled with biotin by Klenow DNA polymerase in the presence of dCTP, dGTP, dTTP, and biotin-14-dATP, followed by ligation using T4 DNA ligase (NEB). After reverse cross-linking, DNA was fragmented by sonication. The DNA fragments were then end-repaired, and the biotinylated DNA fragments were captured using Dynabeads MyOne Streptavidin C1 beads (Invitrogen, Thermo Fisher Scientific). The DNA on beads was ligated to the TruSeq adapters and amplified with PCR for library construction. DNA fragments of 200–500 bp were purified from 1% E-gel and sequenced on Illumina HiSeq in paired-read mode with a read length of 150 nucleotides.

### Hi-C data analysis

Raw sequences were aligned to mm10 genome reference, and genome contact matrices were generated at 5–500 kb resolution used HiC-Pro 3.1.0. Juicer_tools 1.22.01 was applied to convert.pair files to.hic files. Visualization of normalized contact files was performed with Juicebox 2.15. DeepLoop (Zhang et al., 2022) with pre-trained deep-learning models was applied to enhance loop detection. Specifically, HiCorr was used to perform bias correction, which was further input to pre-trained deep enhance model to detect loops.

### CUT&Tag library construction

For Tcf1 and H3K27ac CUT&Tag, cells were sorted following the experimental condition and cell sorting strategy of RNA-seq. For Flag CUT&Tag, cells were sorted following the experimental condition and cell sorting strategy of RIP-seq. 1 × 10^5^ cells were prepared per sample. CUT&Tag was performed according to a standard protocol (Vazyme). In briefly, cells were sorted enriched by ConA-magnetic beads and resuspended in wash Buffer (20 mM HEPES, pH 7.5; 150 mM NaCI, 0.5 mM spermidine; 1× protease inhibitor cocktail; 0.05% digitonin) and then incubated overnight with anti-Tcf1 (1:50, C63D9, cat. no. 2203; Cell Signaling Technology), anti-H3K27ac (1:50, cat. no. ab4729; Abcam), or anti-Flag (1:50, D6W5B, cat. no. 14793; Cell Signaling Technology). The next day, beads were washed in antibody buffer for two times and incubated with anti-rabbit secondary antibody for 1 h at a dilution of 1:100. Then the beads were washed for two times and incubated with proteinA/G-Tnp transposome in Chitag. Next, cells were resuspended in 100 μL tagmentation buffer (10 mM MgCl in Chitag buffer) and incubated at 37°C for 1 h. The tagmentation was terminated by adding 1 μL of 10% SDS at 55°C for 10 min. The DNA fragments added 1 μL spike in (Vazyme) and were extracted by DNA extract beads, then amplified by PCR for 12 cycles with TruePrep index kit for Illumina. Each individual library has been paired-end sequenced on an Illumina NovaSeq platform.

### CUT&Tag data analysis

Raw sequencing data generated from Cut&Tag experiments were first subjected to quality assessment using FastQC. Adapter sequences and low-quality bases were removed using cutadapt. Clean reads were aligned to the mouse reference genome (mm10) using Bowtie2. Peak calling was performed using MACS2 (--nomodel --extsize 272). Peaks with an FDR <0.05 were considered significant. Signal tracks for genome browser visualization were generated using bam2wig, followed by conversion to BigWig format using UCSC tools. These tracks were loaded into IGV for inspection of peak quality and enrichment patterns. For differential binding analysis between experimental groups, read count matrices were constructed using DiffBind (R package), and differentially enriched peaks were identified based on FDR <0.05 and |log_2_ fold change| ≥1 (DESeq2 method). Enrichment signals around transcription start sites (TSS ± 2 kb) were profiled using deepTools (computeMatrix, plotHeatmap).

### Statistical analysis

Data were presented as mean ± SEM. Graphs were produced and statistical analyses were performed in GraphPad Prism 8.0.2 (GraphPad Software, Inc.).

### Online supplemental material

Fig. S1 shows Mettl8 expression in CD8^+^ T cell subsets and generation of Mettl8-tdTomato-Flag mice. Fig. S2 shows T cell maturation in the thymuses and spleens of Mettl8 conditional KO mice. Fig. S3 shows that Mettl8 promotes T_PEX_ differentiation without affecting their proliferation and apoptosis. Fig. S4 shows the expression of effector/exhausted molecules across CD8^+^ T cell subsets. Fig. S5 shows that Mettl8 inhibition promotes CD8^+^ T cell antitumor immunity and synergistically enhances PD-1 blockade.

## Supplementary Material

SourceData F5 is the source file for Fig. 5.

SourceData F6 is the source file for Fig. 6.

SourceData FS5 is the source file for Fig. S5.

## Data Availability

The scRNA-seq data underlying Fig. 1, A–F are openly available in GEO at GSE260448 (Zhang et al., 2024). The scRNA-seq data underlying Fig. 1, G and H are openly available in Zenodo at https://doi.org/10.5281/zenodo.12542577 (Xue and Wu, 2024). The scRNA-seq data underlying Fig. S1, A–E are openly available in GEO at GSE216800 (Zhou et al., 2023). The scRNA-seq data underlying Fig. S1, F–H are openly available in GEO at GSE124691, GSE116390, GSE121478, GSE86028. GSE122713, and GSE137015 (Andreatta et al., 2021). The RNA-seq, m^3^C-seq, RIP-seq, ATAC-seq, Tcf1, Mettl8 and H3K27ac CUT&Tag, and Hi-C data underlying Fig. 4, A–D; and Fig. 5, A, B, D, and E are openly available in GEO at GSE249068. The data underlying Fig. 5 H are openly available in GEO at GSE180094 (Huang et al., 2022).
