# Supplementary figures and images for: Targeting Mettl8-Tcf1 axis promotes CD8+ TPEX differentiation and antitumor immunity
Source: J Exp Med. 2026 Mar 27;223(5):e20250424. doi: 10.1084/jem.20250424 (PMC13023788; doi:10.1084/jem.20250424)

**Fig. 5 F**

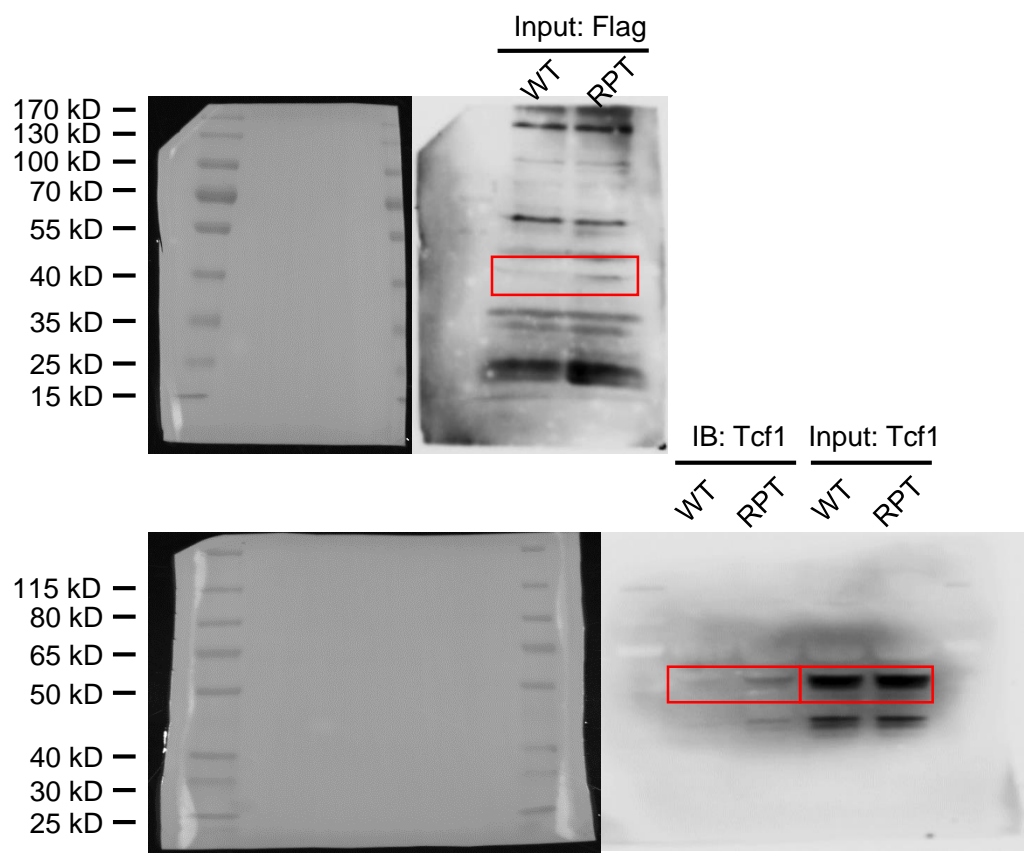

**Fig. 5 G**

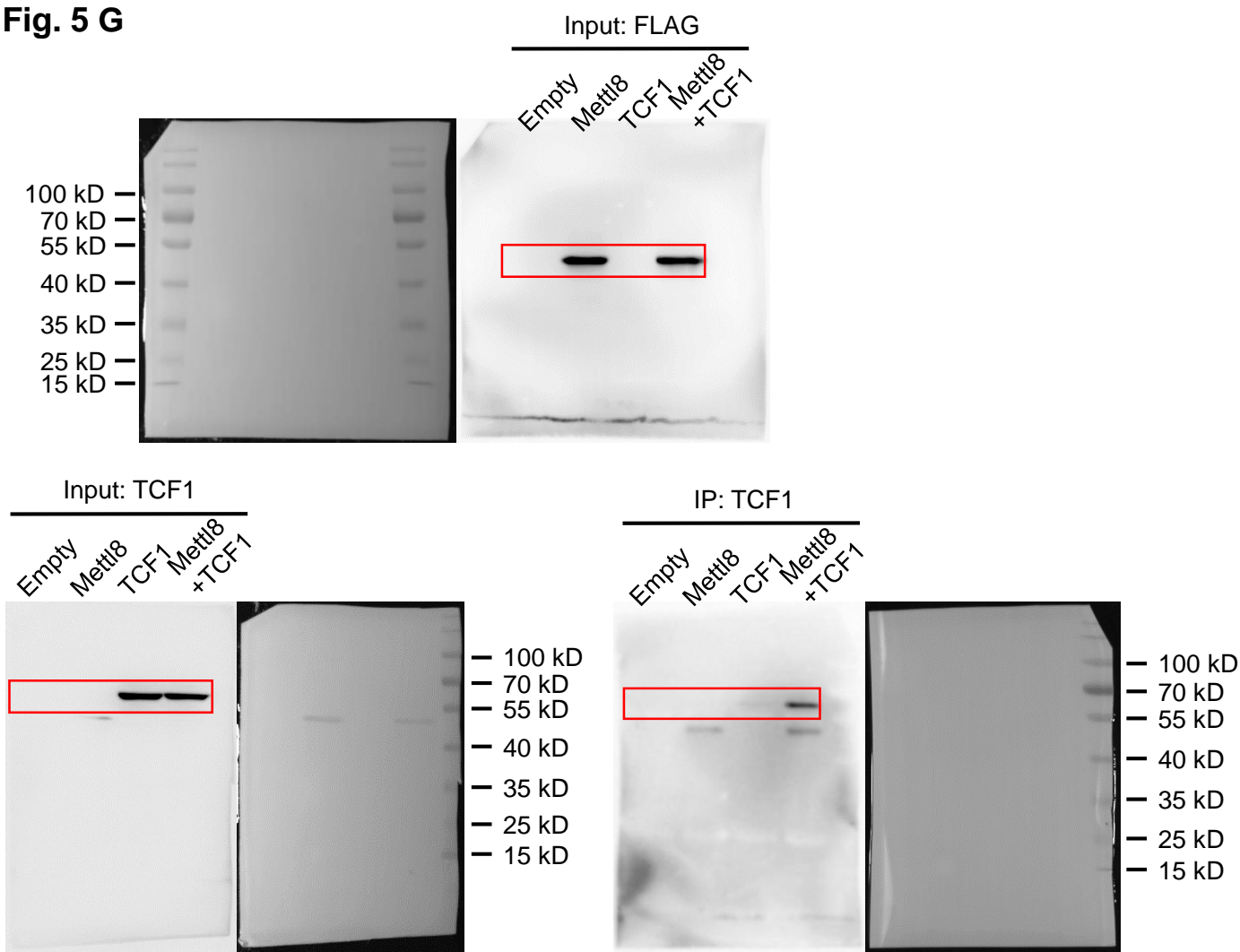

Supplement: SourceData F5 — is the source file for Fig. 5. [file jem_20250424_sourcedataf5.pdf]

**Fig. 6 B**

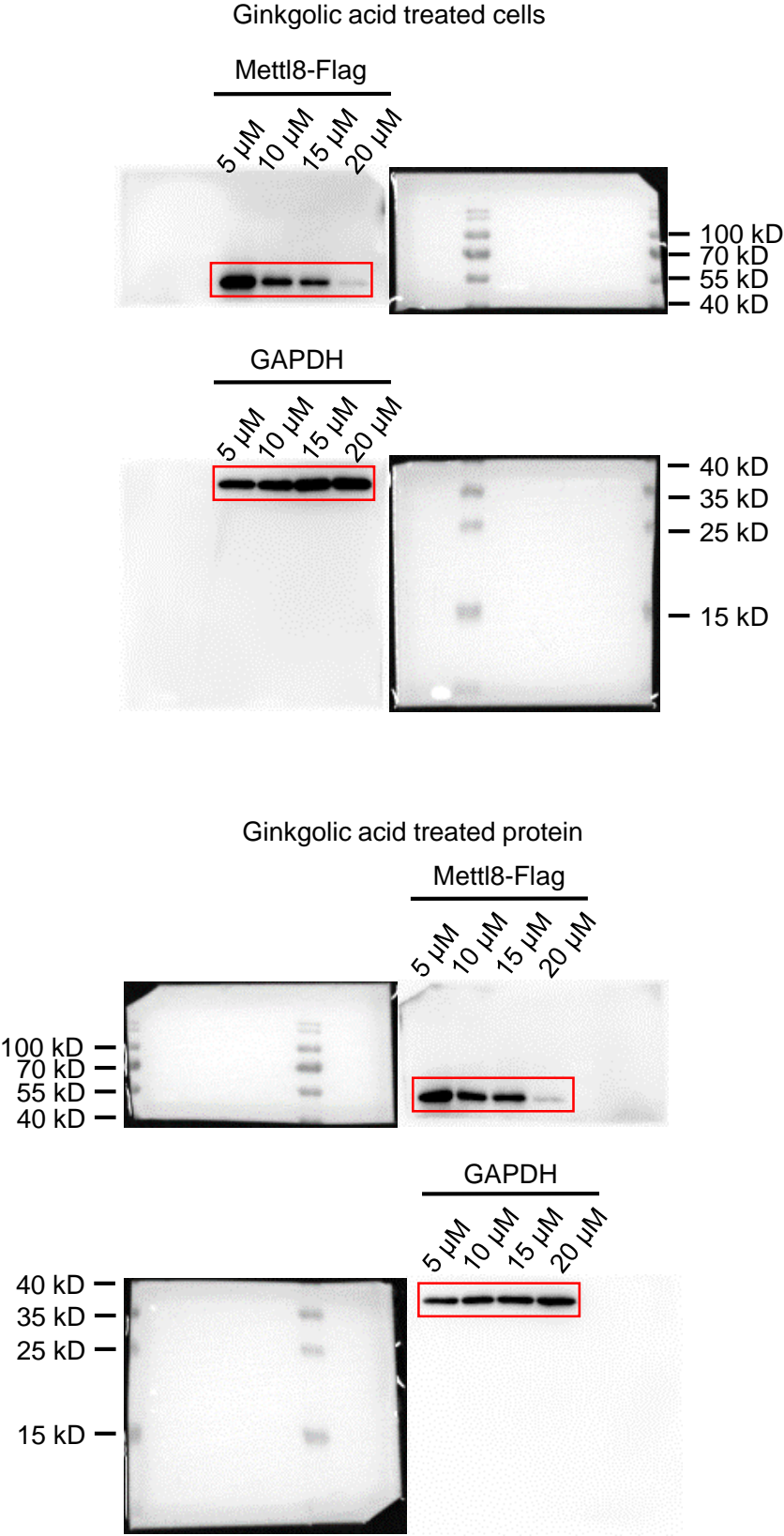

Supplement: SourceData F6 — is the source file for Fig. 6. [file jem_20250424_sourcedataf6.pdf]

Fig. S5 B

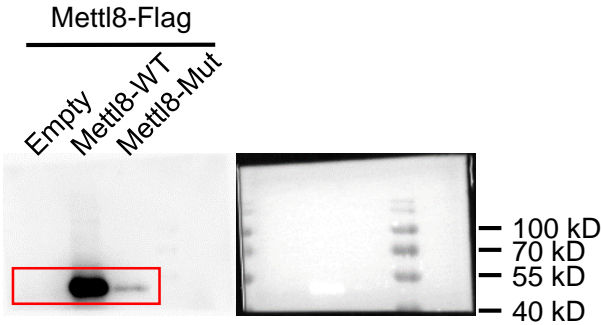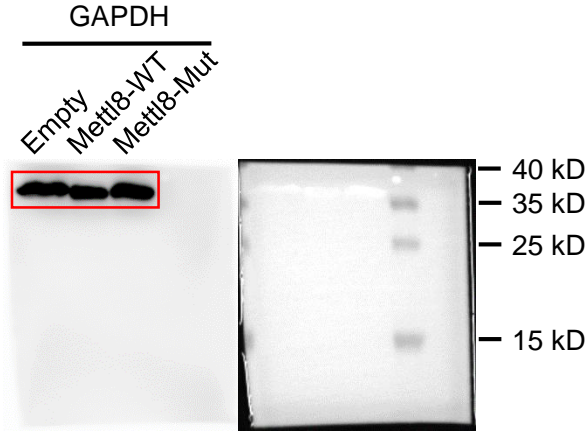

Supplement: SourceData FS5 — is the source file for Fig. S5. [file jem_20250424_sourcedatafs5.pdf]
